# Recurrent inhibition refines mental templates to optimize perceptual decisions

**DOI:** 10.1126/sciadv.ado7378

**Published:** 2024-07-31

**Authors:** Ke Jia, Mengxin Wang, Cecilia Steinwurzel, Joseph J. Ziminski, Yinghua Xi, Uzay Emir, Zoe Kourtzi

**Affiliations:** ^1^ Affiliated Mental Health Center and Hangzhou Seventh People's Hospital, Zhejiang University School of Medicine, Hangzhou 310013, China.; ^2^ Liangzhu Laboratory, MOE Frontier Science Center for Brain Science and Brain-machine Integration, State Key Laboratory of Brain-machine Intelligence, Zhejiang University, Hangzhou 311121, China.; ^3^ NHC and CAMS Key Laboratory of Medical Neurobiology, Zhejiang University, Hangzhou 310058, China.; ^4^ Department of Psychology, University of Cambridge, Cambridge CB2 3EB, UK.; ^5^ Interdisciplinary Institute of Neuroscience and Technology, Key Laboratory for Biomedical Engineering of Ministry of Education, College of Biomedical Engineering and Instrument Science, Zhejiang University, Hangzhou 310027, China.; ^6^ Purdue University School of Health Sciences, West Lafayette, IN 47906, USA.

## Abstract

Translating sensory inputs to perceptual decisions relies on building internal representations of features critical for solving complex tasks. Yet, we still lack a mechanistic account of how the brain forms these mental templates of task-relevant features to optimize decision-making. Here, we provide evidence for recurrent inhibition: an experience-dependent plasticity mechanism that refines mental templates by enhancing γ-aminobutyric acid (GABA)–mediated (GABAergic) inhibition and recurrent processing in superficial visual cortex layers. We combine ultrahigh-field (7 T) functional magnetic resonance imaging at submillimeter resolution with magnetic resonance spectroscopy to investigate the fine-scale functional and neurochemical plasticity mechanisms for optimized perceptual decisions. We demonstrate that GABAergic inhibition increases following training on a visual (i.e., fine orientation) discrimination task, enhancing the discriminability of orientation representations in superficial visual cortex layers that are known to support recurrent processing. Modeling functional and neurochemical plasticity interactions reveals that recurrent inhibitory processing optimizes brain computations for perpetual decisions and adaptive behavior.

## Teaser

Multimodal brain imaging reveals a recurrent inhibitory plasticity mechanism for optimized perceptual decisions.

## INTRODUCTION

Experience and training are known to mold the brain’s structure and functions, facilitating optimal decision-making and skillful actions (*1*, *2*). This experience-dependent plasticity has been shown to extend beyond early development to support the adult brain in translating sensory information to perceptual decisions (*3*, *4*). For example, training is shown to facilitate discriminating fine feature differences (e.g., orientation and motion direction), complex patterns, and objects (*5*, *6*). This ability—known as perceptual learning—is thought to rely on forming mental templates; that is, internal representations of features that are critical for task performance. Training has been suggested to support the brain’s ability to refine these templates and support improved perceptual judgments (*7*–*9*). However, the fine-scale plasticity mechanisms that shape mental templates and support perceptual learning remain largely unresolved.

Computational studies provide some first insights, suggesting that training enhances neural tuning of task-relevant features by altering recurrent connections (e.g., increasing inhibitory connections) in visual cortex (*10*, *11*). Here, we test the hypothesis that γ-aminobutyric acid (GABA)–mediated (GABAergic) inhibition drives this recurrent learning-dependent plasticity. In particular, we test whether long-term training (across 5 days) on an orientation discrimination task boosts perceptual decisions by altering GABAergic inhibition and enhancing representations of task-relevant features (i.e., trained orientations) in superficial V1 layers that are known to be involved in recurrent processing.

Unraveling fine-scale mechanisms of plasticity in the human brain is hampered by the spatial resolution of standard brain imaging techniques. To overcome these limitations, we introduce an ultrahigh-field (UHF; 7 T) multimodal brain imaging approach, combining magnetic resonance spectroscopy (MRS) with functional magnetic resonance imaging (fMRI) at submillimeter resolution. MRS allows us to measure inhibitory (GABA) and excitatory [glutamate (Glu)] neurotransmitter signals noninvasively in the human brain. UHF fMRI allows us to interrogate brain computations at a finer scale and trace brain activity across cortical depths (*12*); that is, middle layers known to be involved in input encoding, superficial layers known to be involved in recurrent processing via horizontal connections, or deeper and superficial layers known to be involved in feedback processing from higher cortical regions (*13*–*16*). We use information-based analyses of fMRI signals across cortical layers to capture mental templates of task-relevant features; that is, fine-tuned feature representations at the scale of multivoxel patterns. This approach allows us to test whether training (i) enhances feature discriminability (i.e., representation distance between trained versus untrained features) or (ii) reduces representation variance across trials.

Our results provide experimental evidence that recurrent inhibition refines mental templates to optimize perceptual decisions. First, we show that training alters orientation-specific representations in superficial (rather than middle or deeper) V1 layers by enhancing the representation distance (i.e., increasing discriminability of trained versus untrained representations) rather than reducing representation variance for the trained orientation. These refined mental templates represent familiar orientations in a fine-tuned manner and relate to improved perceptual discrimination. Second, we demonstrate that training increases GABAergic inhibition—as measured by MRS—in early visual cortex that relates to behavioral improvement. Modeling interactions across multimodal UHF imaging signals provides a mechanistic account of recurrent inhibition that links experience-dependent plasticity across functional and neurochemical levels to adaptive behavior. That is, training alters GABAergic inhibition, enhancing stimulus-specific representations (i.e., representation distance) in superficial V1 layers and unraveling human brain circuit mechanisms for perceptual plasticity and adaptive behavior at unprecedented resolution.

## RESULTS

### Training improves performance in orientation discrimination

**Fig. 1.**
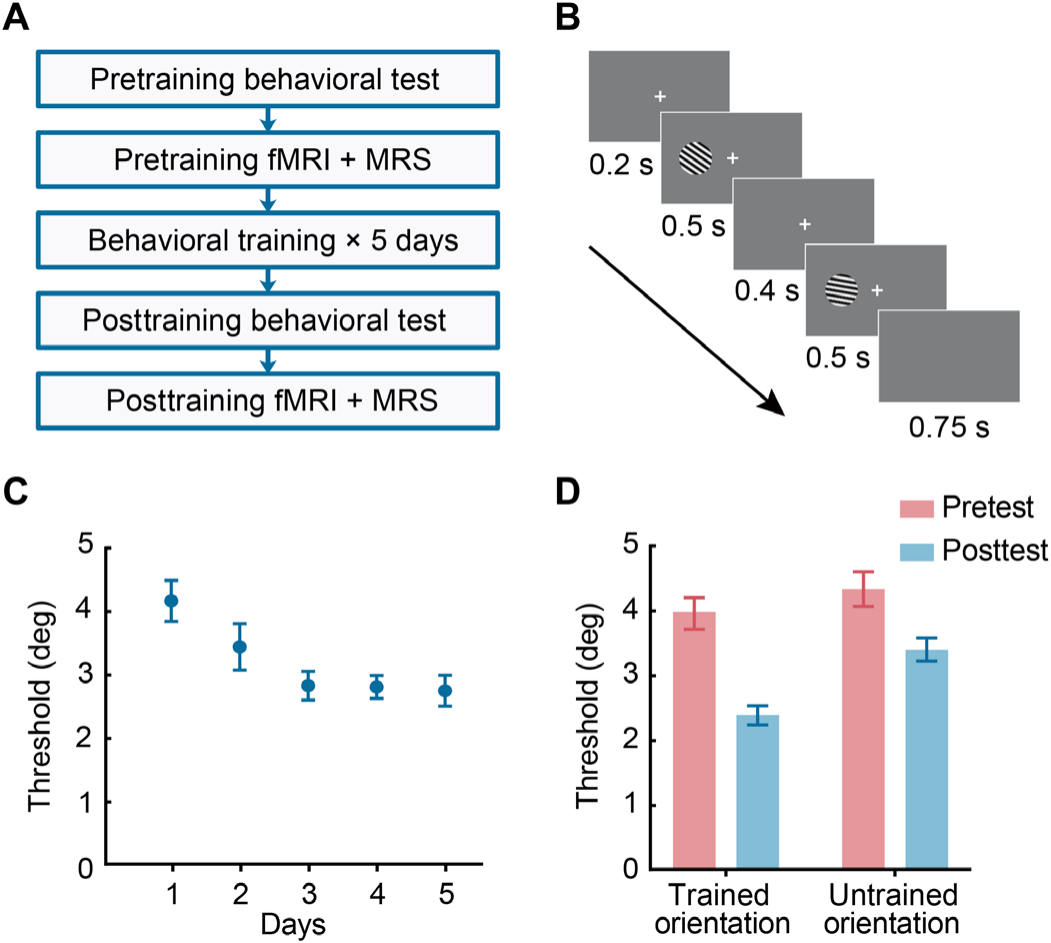
Experimental design, task, and behavioral results. (**A**) Experimental design. Participants were trained on an orientation discrimination task with feedback for five consecutive days. Before and after training, we measured participants’ performance on the same task without feedback during behavioral testing and fMRI scanning. (**B**) Orientation discrimination task. For each trial, participants were asked to report whether the second grating was tilted clockwise or counterclockwise relative to the first grating. (**C**) Mean performance across participants at 79.4% threshold across training sessions. (**D**) Mean threshold performance before and after training. Error bars indicate SEM across participants.

We trained participants (*n* = 29) on an orientation discrimination task (*17*, *18*) for five consecutive days and tested their performance on the same task before and after training (Fig. 1, A and B). Participants’ performance improved during training (Fig. 1C), as indicated by a significant decrease (*t*_28_ = 7.307, *P* < 0.001; paired *t* test) in threshold (79.4% correct using three-down-one-up staircase) after versus before training.

To determine whether the training effect was specific to the trained orientation, we compared participants’ discrimination thresholds for two different orientations (i.e., trained versus untrained orientations that corresponded to 55° or 125°) before and after training. A two-way repeated-measures analysis of variance (ANOVA) (orientation × session) showed a significant two-way interaction (*F*_1,28_ = 12.338, *P* = 0.002; Fig. 1D). Post hoc comparisons showed significant improvement for both the trained (*t*_28_ = 8.914, *P* < 0.001; paired *t* test) and untrained (*t*_28_ = 3.995, *P* < 0.001; paired *t* test) orientations. Further, to quantify behavioral improvement due to training, we calculated a mean percent improvement index (*19*) for each orientation [mean improvement index (MPI) = (pretest threshold − posttest threshold)/pretest threshold × 100%]. We observed that MPI was significantly higher for the trained compared to the untrained orientations (*t*_28_ = 4.986, *P* < 0.001; paired *t* test). Together, these results suggest higher improvement for the trained orientation and partial transfer of learning to the untrained orientation, consistent with previous work [e.g., (*20*)].

### Training enhances stimulus-specific representations in superficial V1 layers

We have previously shown that training results in layer-specific changes in orientation representations in V1; that is, training alters orientation processing in superficial rather than middle or deeper layers of V1 (*21*). However, the mechanisms that underlie this layer-specific perceptual plasticity remain unknown. Here, we ask whether training facilitates perceptual processing in superficial V1 layers by enhancing the stimulus representation (i.e., increased mean representation distance across orientations) (*22*–*24*) or reducing representation variance (i.e., decreased mean distance across blocks for each orientation) (*3*, *25*).

First, we corroborated our previous findings showing enhanced orientation-specific representations in superficial V1 layers after training (*21*), providing a replication in an independent sample (fig. S1). In particular, we used multivoxel pattern analysis (MVPA) to test whether training enhances orientation-specific information across cortical layers. We segmented visual areas by assigning voxels to three layers (superficial, middle, and deeper) using an equivolume approach (Supplementary Text, “Anatomical data analyses” subsection). To improve the spatial specificity of the laminar profiles and control for vasculature-related confounds, we removed voxels that were identified as containing large veins and conducted additional control analyses. Next, we trained linear classifiers to distinguish between (i) trained (55° or 125°) versus control (0°) orientations and (ii) untrained (125° or 55°) versus control (0°) orientations before and after training.

**Fig. 2.**
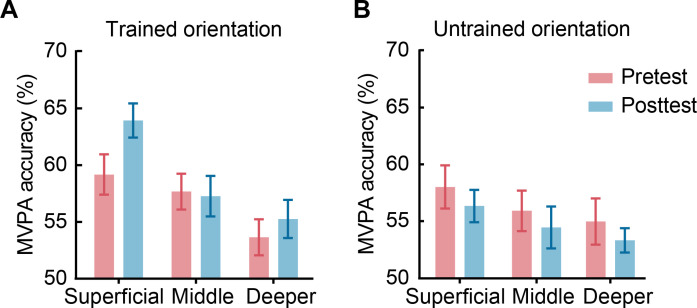
MVPA before and after training across cortical depths in V1. MVPA accuracy (pattern size = 300 voxels) across cortical depths in V1 (superficial, middle, and deeper layers) for the trained (**A**) and untrained (**B**) orientations following correction of vasculature-related effects and unmixing of signals from adjacent layers. Error bars indicate SEM across participants.

Our results demonstrate learning-dependent changes (i.e., increased MVPA accuracy) for the trained orientation in superficial (two-way repeated-measures ANOVA; *n* = 28; session × orientation interaction, *F*_1,27_ = 9.162, *P* = 0.005, permutation test: *P* = 0.001), rather than middle (*F*_1,27_ = 0.531, *P* = 0.473, permutation test: *P* = 0.453) or deeper (*F*_1,27_ = 1.884, *P* = 0.181, permutation test: *P* = 0.185) V1 layers. Post hoc comparisons showed enhanced discriminability (i.e., MVPA accuracy) after versus before training for the trained (*t*_27_ = −2.198, *P* = 0.037, permutation test: *P* = 0.026) compared to the untrained (*t*_27_ = 0.929, *P* = 0.361, permutation test: *P* = 0.368) orientation in superficial layers but not middle (trained orientation: *t*_27_ = 0.159, *P* = 0.875, permutation test: *P* = 0.844; untrained orientation: *t*_27_ = 0.957, *P* = 0.347, permutation test: *P* = 0.386) or deeper (trained orientation: *t*_27_ = −1.011, *P* = 0.321, permutation test: *P* = 0.300; untrained orientation: *t*_27_ = 0.760, *P* = 0.454, permutation test: *P* = 0.422) layers. These results remained significant in superficial layers [Fig. 2; session × orientation (*F*_1,27_ = 6.485, *P* = 0.017, permutation test: *P* = 0.018); trained orientation: *t*_27_ = −2.301, *P* = 0.029, permutation test: *P* = 0.028; untrained orientation: *t*_27_ = 0.715, *P* = 0.481, permutation test: *P* = 0.470] when we unmixed the signal from adjacent layers to control for potential draining vein effects (Supplementary Text, “Correcting for vasculature-related effects” subsection). Further, it is unlikely that these results were confounded by mean normalized fMRI responses (Supplementary Text, “Univariate analysis” subsection) and number of voxels used in the MVPA (fig. S2).

**Fig. 3.**
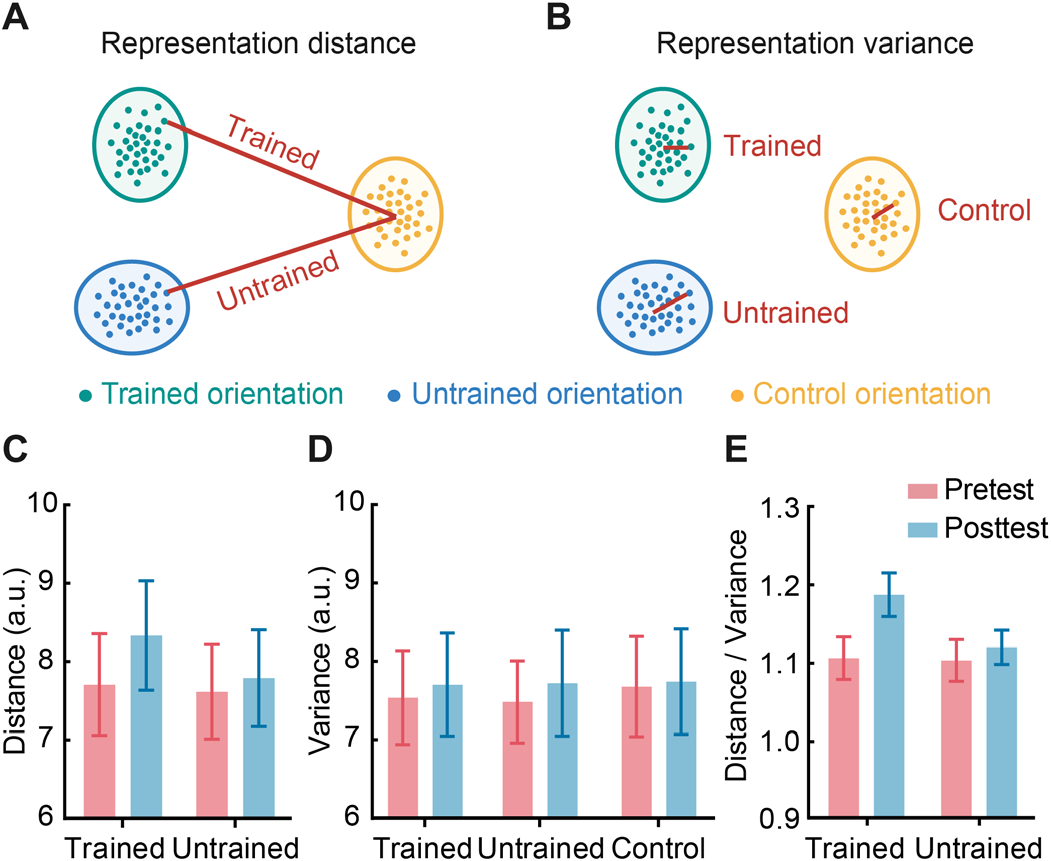
Representation distance and variance analysis. (**A**) Schematic illustration of the representation distance, that is, mean Mahalanobis distance of each block of trained or untrained orientation to the distribution of the control orientation blocks. (**B**) Schematic illustration of the representation variance, that is, mean Mahalanobis distance of each block of trained or untrained orientation to all the other blocks of trained or untrained representations. (**C**) Representation distance (arbitrary unit, a.u.) in superficial V1 layers for the trained and untrained orientations. (**D**) Representation variance (arbitrary unit, a.u.) in superficial V1 layers for the trained, untrained, and control orientations. (**E**) Representation distance/variance in superficial V1 layers for the trained and untrained orientations. Error bars indicate SEM across participants.

Second, we asked whether increased MVPA accuracy for the trained orientation reflects enhanced representation distance (*22*–*24*) or reduced representation variance (*3*, *25*). We estimated the representation distance versus variance in stimulus processing using a Mahalanobis distance analysis. In particular, we estimated the following: (i) representation distance (Fig. 3A), as indicated by cross-condition Mahalanobis distance; that is, mean distance of each block of trained or untrained orientation to the distribution of the control orientation blocks and (ii) representation variance (Fig. 3B), as indicated by within-condition Mahalanobis distances; that is, mean distance of each block of the trained or untrained orientation to all the other blocks of trained or untrained orientations. We reasoned that if training enhances stimulus representation distance, then we would observe increased cross-condition distance between trained and control orientations after training. In contrast, if training reduces representation variance, then we would observe reduced within-condition distance for the trained orientation.

Our results demonstrate that training leads to representation distance enhancement rather than variance reduction in superficial V1 layers. In particular, a two-way repeated-measures ANOVA on representation distance showed a significant session × orientation interaction (*F*_1,27_ = 5.328, *P* = 0.029, permutation test: *P* = 0.025) in superficial (Fig. 3C) but not middle (*F*_1,27_ = 0.077, *P* = 0.783, permutation test: *P* = 0.813) nor deeper (*F*_1,27_ = 0.066, *P* = 0.800, permutation test: *P* = 0.799) V1 layers. Post hoc comparisons showed significantly enhanced representation distance for the trained (*t*_27_ = −2.130, *P* = 0.042, permutation test: *P* = 0.026) compared to the untrained orientation (*t*_27_ = −0.478, *P* = 0.637, permutation test: *P* = 0.622) in superficial V1 layers. In contrast, we did not observe significant changes for representation variance (session × orientation interaction: superficial layers, *F*_1,27_ = 0.121, *P* = 0.730, permutation test: *P* = 0.780, Fig. 3D; middle layers, *F*_1,27_ = 0.193, *P* = 0.664, permutation test: *P* = 0.865; deeper layers, *F*_1,27_ = 0.168, *P* = 0.685, permutation test: *P* = 0.717).

Further, we calculated the ratio of representation distance to variance (distance/variance) for the trained and untrained orientations (i.e., ratio of cross-condition distance to within-condition distance) before and after training. This ratio provides a robust measure of stimulus discriminability, as it indicates the distance between trained (or untrained) and control orientation distributions, taking into account the variability of both orientation (e.g., trained versus control) distributions. A ratio larger than 1 indicates that a given data point for the trained (or untrained) orientation in multidimensional space is closer to the trained (or untrained) orientation distribution compared to the control orientation distribution. A two-way repeated-measures ANOVA on representation distance/variance showed a significant session × orientation interaction in superficial (*F*_1,27_ = 5.645, *P* = 0.025, permutation test: *P* = 0.029) but not middle (*F*_1,27_ = 0.359, *P* = 0.554, permutation test: *P* = 0.536) nor deeper (*F*_1,27_ = 0.361, *P* = 0.553, permutation test: *P* = 0.572) V1 layers (Fig. 3E). Post hoc comparisons showed enhanced representation distance/variance for the trained (*t*_27_ = −2.267, *P* = 0.032, permutation test: *P* = 0.020) compared to the untrained orientation (*t*_27_ = −0.417, *P* = 0.680, permutation test: *P* = 0.672) in superficial V1 layers.

We next estimated the MPI for representation distance, variance, and distance/variance to account for variability in the pretraining data (fig. S3). We observed enhanced distance (trained orientation, *t*_27_ = 2.677, *P* = 0.012; untrained orientation, *t*_27_ = 1.111, *P* = 0.277) and distance/variance (trained orientation, *t*_27_ = 2.556, *P* = 0.017; untrained orientation, *t*_27_ = 0.998, *P* = 0.327) for the trained orientation but no significant changes in representation variance (trained orientation, *t*_27_ = 0.979, *P* = 0.336; untrained orientation, *t*_27_ = 0.915, *P* = 0.368). These results suggest that learning enhances the representation of the trained orientation in superficial V1 layers, independent of any differences in representation variance for different orientations (i.e., trained, untrained, or control orientation). Last, we did not observe any significant differences in representation distance (*t*_27_ = 0.444, *P* = 0.661, permutation test: *P* = 0.676), variance (*t*_27_ = 0.357, *P* = 0.724, permutation test: *P* = 0.864), nor distance/variance (*t*_27_ = 0.108, *P* = 0.915, permutation test: *P* = 0.970) in the pretraining session, suggesting that the learning-dependent changes we observed could not be explained simply by differences before training. Together, our results suggest that training enhances stimulus-specific representations in V1 superficial layers by increasing representation distance between orientations, rather than decreasing representation variance as measured at the scale of submillimeter voxel patterns.

### Training alters GABAergic inhibition in early visual cortex

**Fig. 4.**
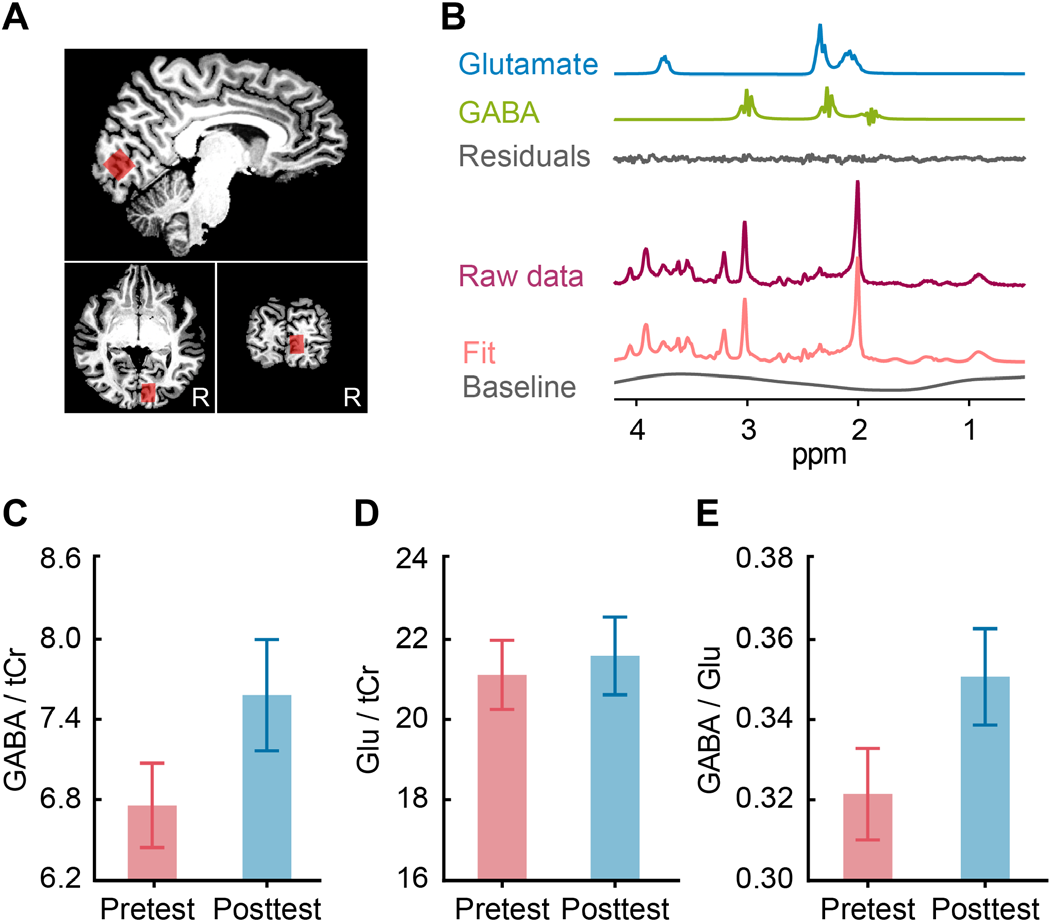
MRS-measured GABA and Glu. (**A**) MRS voxel placement. The MRS voxel was positioned in the right V1 using anatomical landmarks (parallel to the calcarine sulcus) on the acquired T1 scan to ensure that voxel placement was consistent across participants and sessions. (**B**) MRS spectra. Example MRS spectra from early visual cortex for one participant, showing the GABA and Glu fit using LCModel. (**C**) MRS-measured GABA (referenced to tCr) in pretest and posttest sessions. (**D**) MRS-measured Glu (referenced to tCr) in pretest and posttest sessions. (**E**) MRS-measured GABA/Glu in pretest and posttest sessions. Error bars indicate SEM across participants.

Using MRS measurements of GABA and Glu, we demonstrate that training alters inhibition processes in early visual cortex (Fig. 4, A and B). In particular, training significantly increased GABA [referenced to total creatine (tCr): *t*_24_ = −2.134, *P* = 0.043, paired *t* test; Fig. 4C] but did not significantly change Glu (*t*_24_ = −0.689, *P* = 0.497, paired *t* test; Fig. 4D) concentrations in early visual cortex. Note that limitations in MRS spatial resolution and the MRS voxel placement result in differences in the coverage of the MRS voxel in relation to the V1 region of interest (ROI). To relate MRS measurements to V1 processing, for each participant, we normalized GABA and Glu concentrations to the spatial overlap of the MRS voxel with the V1 ROI.

We next tested for learning-dependent changes in the MPI for GABA and Glu to account for variability in the pretraining data. We observed similar results; that is, learning-dependent increase in GABA (*t*_24_ = 2.355, *P* = 0.027) but not Glu (*t*_24_ = 0.970, *P* = 0.342). These learning-dependent changes in GABA remained significant when we (i) referenced GABA and Glu to water (GABA: *t*_24_ = 2.098, *P* = 0.047; Glu: *t*_24_ = 0.078, *P* = 0.939); (ii) controlled for voxel tissue composition using alpha correction (GABA: *t*_24_ = 2.314, *P* = 0.030; Glu: *t*_24_ = 0.965, *P* = 0.344); (iii) or by dividing GABA and Glu concentration by 1–fCSF (fraction of cerebrospinal fluid) (GABA: *t*_24_ = 2.334, *P* = 0.028; Glu: *t*_24_ = 1.001, *P* = 0.327). Note that no significant differences were observed across sessions in data quality measures or the overlap between MRS voxel and V1 ROI (fig. S4 and table S1). Further, the learning-dependent changes we observed in GABA are unlikely to reflect variations in attention to the task, as the task performance was maintained at ~79.4% across sessions, using staircase-based training.

Further, previous studies and computational models have suggested that both inhibitory and excitatory processes contribute to orientation selectivity in V1 (*10*, *26*, *27*). In particular, GABA/Glu has been shown to relate to behavioral improvement in visual discrimination tasks (*28*, *29*). In light of this previous work, we computed MPI of GABA/Glu ([posttraining ratio − pretraining ratio]/ pretraining ratio × 100%) to capture the role of both inhibitory and excitatory neurotransmitters in learning. We observed significantly higher GABA/Glu after training (*t*_24_ = 2.213, *P* = 0.037; Fig. 4E), suggesting that training alters inhibition/excitation in early visual cortex.

Last, we conducted a control experiment measuring MRS GABA and Glu concentrations in early visual cortex in two sessions (pretest and posttest) without behavioral training in between. Our results did not show significant changes between sessions (GABA: *t*_8_ = −1.094, *P* = 0.306; Glu: *t*_8_ = −0.781, *P* = 0.457; GABA/Glu: *t*_8_ = −1.078, *P* = 0.313; paired *t* test), suggesting that the increase we observed in GABA after training could not be simply due to repeated measurements over time. This is consistent with our previous findings showing learning-dependent changes in MRS-GABA within (*30*) and across (*31*) sessions, compared to lack of significant changes between MRS measurements when no training is involved.

### Linking functional and neurochemical plasticity to behavioral improvement

**Fig. 5.**
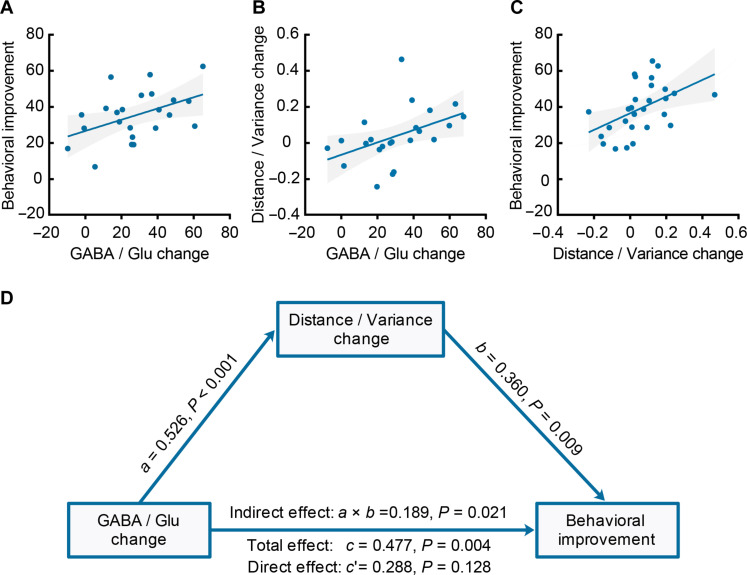
Linking functional and neurochemical plasticity to behavioral improvement. (**A**) Skipped Pearson’s correlation showing a significant positive correlation of GABA/Glu change in early visual cortex with behavioral improvement. (**B**) Skipped Pearson’s correlation showing a significant positive correlation of GABA/Glu change with representation distance/variance change in V1 superficial layers. (**C**) Skipped Pearson’s correlation showing a significant positive correlation of representation distance/variance change in V1 superficial layers with behavioral improvement. (**D**) Mediation analysis showing that increased GABA/Glu drives behavioral improvement by enhancing the discriminability of the trained stimulus in superficial V1 layers.

We next asked whether functional and neurochemical plasticity mechanisms interact to predict behavioral improvement. First, following previous work (*28*, *29*), we tested the relationship between learning-dependent changes in GABA/Glu and behavioral improvement. Pearson correlation analyses showed a significant positive relationship between learning-dependent changes in behavior and GABA/Glu (*r* = 0.456, *P* = 0.029, two bivariate outliers) after controlling for the overlap between the MRS voxel and the V1 ROI (Fig. 5A). This positive relationship remained significant when we (i) controlled for behavioral improvement for the untrained orientation (*r* = 0.486, *P* = 0.022), (ii) controlled for behavioral performance in the pretraining session (*r* = 0.604, *P* = 0.004), and (iii) tested for correlation with GABA rather than GABA/Glu (*r* = 0.485, *P* = 0.026). In contrast, we did not observe significant correlations between GABA changes and behavioral improvement for the untrained orientation (*r* = 0.289, *P* = 0.181).

Second, we show that learning-dependent changes in GABA/Glu relate to layer-specific changes in orientation-specific representations in V1. In particular, Pearson correlation analyses showed a significant positive relationship between learning-dependent changes in GABA/Glu and representation distance/variance in superficial V1 layers (*r* = 0.580, *P* = 0.005, one bivariate outlier) after controlling for variability in the overlap between the MRS voxel and the V1 ROI across participants, and the representation changes for the untrained orientation. This correlation remained significant when we (i) controlled for changes in representation distance/variance in middle (*r* = 0.447, *P* = 0.042) or deeper (*r* = 0.579, *P* = 0.006) layers, (ii) tested for correlation with representation distance rather than distance/variance (*r* = 0.552, *P* = 0.008), and (iii) tested for correlation with changes in GABA rather than GABA/Glu (*r* = 0.462, *P* = 0.030). These results suggest a strong link between enhancement of orientation-specific representations in superficial layers and GABAergic inhibition.

Third, we show that learning-dependent changes in orientation-specific representations in superficial V1 layers relate to behavioral improvement. In particular, Pearson correlation analyses showed a significant positive relationship of changes in representation distance/variance with behavioral improvement for the trained orientation in superficial (*r* = 0.393, *P* = 0.047) but not middle (*r* = 0.165, *P* = 0.422) nor deeper (*r* = 0.061, *P* = 0.768) layers, after controlling for changes in the untrained orientation. Further, the positive relationship remained significant when we (i) controlled for behavioral performance in the pretraining session (*r* = 0.411, *P* = 0.041) and (ii) tested for correlation with representation distance rather than distance/variance (*r* = 0.401, *P* = 0.047). In contrast, we did not observe significant correlations between behavioral improvement for the untrained orientation and changes in representation distance/variance for the trained orientation (superficial V1 layers: *r* = 0.176, *P* = 0.388).

Further, to test whether the changes we observed were due to training rather than potential differences in measurements across sessions (e.g., related to MRI data quality or participant state), we calculated a learning modulation index (LMI) that contrasts differences across sessions for the trained against the untrained orientation. In particular, LMI for representation distance/variance was calculated as: [posttest ratio for trained orientation − pretest ratio for trained orientation] − [posttest ratio for untrained orientation − pretest ratio for untrained orientation] (*17*). Note that LMI extends beyond MPI that captures only differences across sessions for the trained orientation. We observed a significant positive relationship between learning-dependent changes in LMI for representation distance/variance in superficial V1 layers and GABA/Glu (Fig. 5B; *r* = 0.452, *P* = 0.030, one bivariate outlier) after controlling for variability in the overlap between the MRS voxel and the V1 ROI across participants. This relationship remained significant when we tested for correlation with LMI for distance (*r* = 0.449, *P* = 0.031) rather than distance/variance. Further, we observed a significant positive relationship of LMI for distance/variance with behavioral improvement (Fig. 5C) in superficial (*r* = 0.481, *P* = 0.010) but not middle (*r* = 0.052, *P* = 0.791) nor deeper (*r* = 0.228, *P* = 0.243) layers. This relationship remained significant when we (i) tested for correlation with LMI for distance (*r* = 0.411, *P* = 0.030) rather than distance/variance and (ii) controlled for behavioral improvement for the untrained orientation (*r* = 0.428, *P* = 0.026).

Last, mediation analysis showed that learning-dependent changes in GABAergic inhibition drive behavioral improvement by enhancing orientation-specific representations in V1 superficial layers. In particular, mediation analysis (GABA/Glu; distance/variance in V1 superficial layers, behavioral improvement) showed a significant total effect *c* = 0.477, *z* = 2.879, *P* = 0.004, confidence interval (CI) = [0.152, 0.802] due to an indirect significant effect of distance/variance (*ab* = 0.189, *z* = 2.316, *P* = 0.021, CI = [0.029, 0.350]). No significant direct effect of GABA/Glu change to behavioral improvement was observed (*c′* = 0.288, *z* = 1.523, *P* = 0.128, CI = [−0.082, 0.658]) (Fig. 5D). Further, the mediation effect remained significant when we (i) tested for GABA rather than GABA/Glu (*ab* = 0.150, *z* = 2.299, *P* = 0.021, CI = [0.022, 0.278]) and (ii) controlled for behavioral improvement for the untrained orientation (*ab* = 0.191, *z* = 2.152, *P* = 0.031, CI = [0.017, 0.365]). The mediation effect was not significant for middle (*ab* = −0.005, *z* = −0.100, *P* = 0.920, CI = [−0.102, 0.092]) or deeper (*ab* = −0.018, *z* = −0.221, *P* = 0.825, CI = [−0.176, 0.141]) V1 layers. These results suggest a key role of GABAergic inhibition in enhancing orientation-specific representations in superficial layers in primary visual cortex for improved fine discriminations; that is, increased inhibition drives behavioral improvement by enhancing the discriminability of the trained orientation in superficial visual cortex layers.

## DISCUSSION

We propose a recurrent inhibition plasticity mechanism that refines task-relevant feature templates to support our ability for optimized perceptual decisions through training. In particular, we use an UHF multimodal brain imaging approach to investigate at unprecedented resolution the interactions of neurochemical and functional plasticity mechanisms that support our ability to translate sensory information to perceptual decisions. First, we leverage the submillimeter resolution of 7-T laminar fMRI to interrogate plasticity mechanisms across cortical depths that are known to be associated with dissociable neural computations. Our findings provide evidence for recurrent experience-dependent plasticity that amplifies the representation distance between orientations in V1 superficial layers enhancing the discriminability of trained orientations, rather than reducing representation variance. Second, we demonstrate that training results in increased GABAergic inhibition—as measured by MRS—in early visual cortex that relates to behavioral improvement. Modeling neurochemical and functional plasticity interactions reveals that training alters GABAergic inhibition in visual cortex that drives improved perceptual judgments by strengthening orientation-specific representations (i.e., discriminability of the trained orientation as indicated by representation distance) in superficial V1 layers. Together, our findings provide evidence for recurrent inhibition as an integrative experience-dependent plasticity mechanism that optimizes the neural code for perceptual decisions.

First, previous studies have shown that training enhances the representation of task-relevant features at the level of neural populations (*21*, *24*, *32*). Extending beyond this work, we have recently shown that training alters orientation-specific representations in superficial layers of primary visual cortex (*21*), suggesting that training alters recurrent processing rather than local information encoding or feedback from higher decision-related regions. However, the mechanisms underlying this recurrent functional plasticity remain unknown. Here, we combine UHF fMRI with information-based analysis (i.e., multivoxel pattern classification) to test competing hypotheses; that is, training (i) enhances distinctive representations for the trained compared to untrained orientations as measured at the level of large neural populations by multivoxel patterns and (ii) decreases the variance in orientation-specific representations. We corroborate our previous results, showing learning-dependent changes in superficial—rather than middle or deeper—V1 layers for the trained compared to untrained orientations in an independent sample. Extending beyond this finding, we provide evidence that this recurrent functional plasticity relates to enhanced representation distance for the trained orientation in the multivoxel pattern space, consistent with previous neurophysiology studies showing that signal enhancement, rather than internal noise reduction, plays a key role in perceptual learning (*23*, *24*). Further, we demonstrate that these refined orientation-specific representations relate to behavioral improvement, consistent with the role of training in reweighting sensory information to optimize perceptual decisions (*22*, *33*–*35*).

Second, we test the role of GABAergic inhibition in driving layer-specific functional plasticity and shaping orientation-specific representations. Our results demonstrate increased GABAergic inhibition in early visual cortex following extensive (over 5 days) discrimination training. Further, we demonstrate that increased GABA relates to improved perceptual judgments, consistent with previous studies linking GABAergic inhibition to performance and learning in perceptual (visual discrimination) and motor tasks (*30*, *36*–*41*). Note that MRS captures neurochemical plasticity related to the visual discrimination task before and after training rather than specifically to the trained orientation. Recent developments in functional MRS may provide further insights into stimulus-specific GABAergic inhibition (i.e., trained versus untrained orientation) (*42*, *43*).

Third, we provide evidence that GABAergic plasticity shapes layer-specific functional plasticity. Combining MRS with UHF fMRI suggests that GABAergic inhibition drives improved perceptual decisions by enhancing orientation-specific representations in superficial V1 layers. Note that methodological limitations (i.e., coarser MRS spatial resolution compared to fMRI) mean that it is not possible to estimate neurotransmitter concentration across cortical layers using MRS. Despite this limitation, mediation analysis showed that learning-dependent changes in visual cortex inhibition/excitation predict enhanced representation distance for the trained orientation in superficial—rather than middle or deeper—V1 layers. These GABAergic-driven changes in recurrent visual processing that are primarily associated with superficial V1 layers propose a recurrent inhibition mechanism of learning-dependent plasticity for optimizing perceptual judgments. Future advances in magnetic resonance spectroscopic imaging may afford higher spatial resolution and support a tighter link between MRS and fMRI signals.

Our results are consistent with previous neurophysiological studies linking GABAergic inhibition (*44*) and interneurons (please cite the paper in the comments section here) to cortical tuning and pharmacological interventions showing that GABA agonists enhance orientation selectivity in the visual cortex, while blocking GABAergic inhibition results in broader neural tuning (*45*, *46*). Further, previous studies have suggested that learning-dependent changes in superficial V1 layers are due to cross-orientation inhibition (*10*, *21*), that is, suppression of neurons that are selective for similar orientations across columns. Cross-orientation inhibition is shown to be more pronounced in superficial layers and support orientation tuning via horizontal connections between V1 columns (*47*–*50*). Thus, training may enhance neural tuning by inhibiting orientations close to the trained orientation in superficial V1 layers. These results in support of recurrent inhibitory processing via horizontal connections are consistent with computational modeling proposing that training sharpens neural tuning by modifying recurrent connections (e.g., increasing inhibitory connections) near the trained orientation.

It is important to note that our MRS measurements captured GABAergic inhibition before versus after training rather than during learning. Understanding the dynamics of inhibition and excitation during learning and how they contribute to behavioral improvement remains an open question. Previous imaging studies showed that training and overtraining changes GABA/Glu in the short term, suggesting increased GABAergic inhibition early in the training that then returns to baseline levels within hours (*28*, *29*). Recent neurophysiological studies showed that longer-term training (over 9 days) on a discrimination task resulted in increased stimulus selectivity in parvalbumin GABAergic interneurons and pyramidal cells, suggesting that changes in activity of GABAergic interneurons is observed following consolidation of learning (i.e., after behavioral performance has saturated, similar to our findings) (*51*, *52*). Future work, introducing measurements during training (i.e., shorter- versus longer-term timescales), is needed to understand the dynamics of recurrent inhibition during learning.

In sum, our results provide evidence for a recurrent inhibitory plasticity mechanism for perceptual learning. Combining multimodal UHF brain imaging with information-based analysis, we reveal a key role for recurrent inhibition in refining information processing for optimized perceptual decisions. Training refines mental templates by fine-tuning the representation of task diagnostic features (i.e., trained orientation), suppressing similar orientations across cortical columns via horizontal connections in superficial layers of primary visual cortex. Uncovering this multimodal plasticity mechanisms at the intersection of neurochemical and functional signals provides insights in bridging the knowledge gap between animal and human brain circuits that support learning and adaptive behavior.

## MATERIALS AND METHODS

### Experimental design

#### Participants

Thirty participants (mean age, 22.47 years and SD, 3.27 years) took part in the study. Data from one participant was excluded because of technical problems during data acquisition. All participants were right-handed, had normal or corrected-to-normal vision, were not under any prescription medication, and gave written informed consent. Participants were naive to the aim of the study and received payment for their participation. All experiments were approved by University of Cambridge Ethics Committee (PRE.2017.057).

#### Stimuli

Stimuli comprised oriented sinusoidal gratings that were presented at an eccentricity of 5° against a uniform gray background. Gratings of random phase had a fixed diameter of 4°, contrast of 0.8, and spatial frequency of 1 cycle/deg. The contrast decreased to zero over the outer 0.5° radius of the gratings. The stimuli were presented in the left visual field, as data were collected from a unilateral MRS voxel in the right hemisphere.

#### Experimental procedure

The study comprised a pretest (two sessions, one behavioral test, and one fMRI test), a training (five sessions), and a posttest (two sessions, one behavioral test, one fMRI test) phase (Fig. 1A). Each session was completed on a separate day. Participants performed a two-interval forced choice orientation discrimination task. Participants’ performance in the task was measured using a three-down-one-up staircase with 15 reversals converging at 79.4% performance. We trained participants with feedback on the orientation discrimination task presenting gratings at the same orientation and location and tested the participants without feedback. Before and after training in the laboratory, participants performed the orientation discrimination task during functional MRI and MRS data acquisition without feedback.

Functional scans were acquired using a two-dimensional gradient-echo echo-planar imaging (GE-EPI) sequence (*53*) at submillimeter resolution (0.8 mm isotropic) and field of view covering occipitotemporal and posterior areas. MRS data were acquired using a semilocalization by adiabatic selective refocusing (semi-LASER) sequence. The MRS voxel (15 mm isotropic) was positioned in right early visual cortex, parallel to the calcarine sulcus, retinotopically mapped with the stimulus location (i.e., left visual field), avoiding proximity to the dura to minimize macromolecule contamination. To ensure consistent voxel placement across sessions and participants, the MRS voxel was manually positioned on the basis of each participant’s T1w anatomical image using anatomical landmarks (e.g., calcarine sulcus). Voxel position was similar across sessions (mean absolute difference in position between voxel center, X: M = 0.68 mm, SD = 0.68 mm; Y: M = 1.25 mm, SD = 1.15 mm; Z: M = 1.14 mm, SD = 0.72 mm). The mean gray matter (GM) tissue fraction for pretraining and posttraining was 41.61 and 41.30%. GM tissue content did not differ significantly between sessions, paired *t* test, *t*_23_ = 0.378, *P* = 0.709.

#### Statistical analysis

Repeated-measures ANOVA was used to assess differences across conditions, for behavioral, fMRI (MVPA), and MRS data. For fMRI (MVPA) data, ANOVA results were corroborated by permutation tests that have been shown to be more appropriate for comparing classification accuracy across conditions (*54*, *55*). We evaluated correlations between fMRI, MRS, and behavioral indices using Pearson’s correlation after outlier exclusion [bivariate outliers were identified using the Robust Correlation Toolbox (*56*)]. In particular, bivariate outliers were detected using the box-plot rule on *z*-scored values: The algorithm calculates orthogonal distances of all data points from the center of the bivariate distribution and marks as outliers data points with distances that exceed the interquartile range (*37*). Correlation and mediation analysis was conducted with JASP v0.17.1.

## Supplementary Materials

### This PDF file includes:

Supplementary Text Figs. S1 to S4 Tables S1 and S2 References
