# Supplementary material for: Recurrent inhibition refines mental templates to optimize perceptual decisions
Source: Sci Adv. 2024 Jul 31;10(31):eado7378. doi: 10.1126/sciadv.ado7378 (PMC11290482; doi:10.1126/sciadv.ado7378)
Supplement: Supplementary file 1 — Supplementary Text Figs. S1 to S4 Tables S1 and S2 References [file sciadv.ado7378_sm.pdf]

Science Advances
AAAS

Supplementary Materials for

Recurrent inhibition refines mental templates to optimize perceptual decisions

Ke Jia *et al.*

Corresponding author: Zoe Kourtzi, zk240@cam.ac.uk; Ke Jia, kjia@zju.edu.cn

Sci. Adv. **10**, eado7378 (2024)
DOI: 10.1126/sciadv.ado7378

This PDF file includes:

Supplementary Text
Figs. S1 to S4
Tables S1 and S2
References

Supplementary Text

Participants

Thirty participants (mean age: 22.47 years and SD: 3.27 years) took part in the study. Data from one participant was excluded due to technical problems during data acquisition. All participants were right-handed, had normal or corrected-to-normal vision, were not under any prescription medication and gave written informed consent. Participants were naive to the aim of the study and received payment for their participation.

We only included male participants in our study due to the potential influence of menstrual cycle on GABA measurements (*31*). Extensive research has been conducted on this topic (*57–62*) with several studies restricting MRS-GABA investigations to males (*63–71*). Key hormones, such as estrogen and progesterone have been found to have suppressive or facilitatory effect on GABA transmission (*58*, *59*, *72*), which may confound within-subject GABA measurements over time. However, developing precise methods to control for the effects of the menstrual cycle on MRS GABA measurements is challenging due to the physiological complexity (i.e., phase and regional effects of menstrual cycle on GABA) and limited knowledge of the kinetics of menstrual cycle-related GABA changes in humans. The phase and regional effects of the menstrual cycle on GABA, as well as the kinetics of GABA changes across different menstrual cycle days, are likely to vary significantly among participants. Since our study involves repeated MRS GABA measurements over time, it is not feasible to adequately control for menstrual cycle effects. To address these limitations and enhance generalizability, ongoing large-scale multi-site studies are expected to provide normative data and will advance our understanding of these cyclical effects. These studies will also enable the development of precise control methodologies and allow for the inclusion of female participants.

Experimental Design and Procedure

The study comprised a pre-test (2 sessions, 1 behavioral test, 1 fMRI test), a training (5 sessions) and a post-test (2 sessions, 1 behavioral test, 1 fMRI test) phase (Fig. 1A). Each session was completed on a separate day.

We employed a two-interval forced choice (2IFC) orientation discrimination task. Each trial began with a fixation cross for 200 ms followed by the sample and test gratings that were presented sequentially for 500 ms each and separated by a 400 ms inter-stimulus interval (ISI). Participants were asked to fixate and report (by key press) within 1250 ms after the onset of the test grating whether it was titled clockwise or counter-clockwise relative to the sample stimulus.

Experiments were controlled using MATLAB and Psychophysics toolbox 3.0 (*73*, *74*). For the behavioral sessions, stimuli were presented on a 21-inch CRT monitor (1600 × 1200 pixel resolution, 85 Hz frame rate) at a distance of 110 cm. Gamma correction was applied to the monitor. For the fMRI scans, stimuli were presented using a projector and a mirror setup (1024 × 768 pixel resolution, 100 Hz frame rate) at a viewing distance of 110 cm. Angular stimulus size was the same across behavioral and fMRI sessions.

Participants' performance in the task was measured using a 3-down-1-up staircase with 15 reversals converging at 79.4% performance. A fixed step size of 0.25° was used for each reversal. The reference orientation for the trained and untrained stimuli was 55° or 125°. We added a uniformly distributed random jitter within ±5° to the reference orientation across trials to ensure that participants compared two gratings in each trial, rather than the test grating to a fixed reference orientation. The training reference orientation (55° vs. 125°) was counterbalanced across participants. Participants were tested with a control orientation (0°, vertical) that differed equally from the trained and untrained orientation (55° or 125°). This allowed us to test orientation-specific

pattern changes in fMRI signals due to training (i.e., learning-dependent changes to the trained vs. untrained orientation), by comparing separately the trained vs. the untrained orientations to the control orientation (*21*).

Behavioral Tests. To familiarize participants with the task before testing, each participant performed a 30-trial practice run (10 trials per condition, i.e., three different reference orientations) using a fixed above-threshold angle difference (8°). For both the pre- and post-training test, participants performed the orientation discrimination task for 6 test staircase runs (2 runs per condition in random order). For each condition, the starting angle difference between sample and test stimulus for the first run was 5°. For the second run, the starting angle difference was determined by the threshold in the preceding run. The discrimination threshold for each condition was the mean threshold across two runs. No feedback was provided to the participants during the test phase.

Behavioral Training (5 sessions). We trained participants on the orientation discrimination task (16 staircases per session, ~1 h) with gratings presented at the same orientation and location throughout training. The starting angle difference between sample and test stimuli for the first staircase of the first training session was 5°. For the remaining staircases, the starting angle difference was determined by the threshold of the preceding staircase. Training orientation (i.e., 55° vs. 125°) were counterbalanced across participants. Participants were given auditory error feedback per trial. For one participant, only 4 training sessions were completed due to time constraints; for another participant, an extra training session was completed due to delay in the post-training scanning session.

fMRI sessions. Before and after training in the lab, participants completed 5-6 runs of the orientation discrimination task during scanning. For each participant, we also collected data from an anatomical scan.

For the orientation discrimination task, each run started with a fixation block (8.24 s) followed by one stimulus block for each of the three conditions (14.42 s) comprising gratings presented at the trained, untrained or control orientation. There was a fixation period of 10.3 s between each stimulus block. The order of orientations was randomized across the three stimulus blocks. This sequence of fixation and stimulus blocks was repeated six times in each run. For each stimulus block, participants completed six trials of the orientation discrimination task. The task parameters (i.e., sample and test duration) were the same as for the behavioral tests and no feedback was provided to the participants. The fixed angle difference between sample and test stimuli for each condition was determined by the preceding behavioral session. This allowed us to match task difficulty (~79.4% correct) before and after training.

Data acquisition

MRI data acquisition. Imaging data were acquired at the Wolfson Brain Imaging Centre, University of Cambridge, on a Siemens 7T Terra scanner with a 32-channel phased-array head coil (Nova Medical, Inc., Wilmington, MA, USA). For each participant, anatomical images were acquired using MP2RAGE T1-weighted sequence (TR = 5000 ms, TE = 2.56 ms, FOV = 208 × 208 mm^2, resolution: 0.65 × 0.65 × 0.65 mm^3, number of slices: 240, slice orientation: Sagittal). Functional scans were acquired using a 2D Gradient Echo, Echo Planar Imaging (GE-EPI) sequence (*53*) (TR = 2060 ms, TE = 26.4 ms, FOV = 148 × 148 mm^2, flip angle: 70°, resolution: 0.8 × 0.8 × 0.8 mm^3, number of slices: 56, partial Fourier = 6/8, GRAPPA factor = 3, Multi-Band

factor = 2, bandwidth = 1034 Hz/Pixel, echo spacing = 1.09 ms). The field of view covered occipito-temporal and posterior areas; manual shimming was performed prior to the acquisition of the functional scans.

MRS data acquisition. MRS data were acquired using a semi-localization by adiabatic selective refocusing (semi-LASER) sequence (128 averages, TR = 5000 ms, TE = 36 ms). We chose to utilize a short-echo, full signal intensity semi-LASER sequence to achieve lower apparent T2 relaxation, minimal J-coupling evolution, and smaller chemical shift displacement errors relative to the PRESS and STEAM sequences (*75*). In addition, the adiabatic refocusing pulses in the semi-LASER provided minimal signal loss, high B1+ insensitivity and localization against the varying destructive interferences throughout the brain at ultra-high field. This MRS sequence has been extensively tested and resulted in high-quality spectra across high and ultra-high field magnetic fields at different MRI centers (*39*, *43*, *75–79*). We used the VAPOR water suppression (*80*) and the outer volume suppression (*78*).

The MRS voxel ($15 \times 15 \times 15$ mm^3 isotropic) was positioned in right early visual cortex, parallel to the calcarine sulcus, retinotopically mapped with the stimulus location (i.e., left visual field), avoiding proximity to the dura to minimise macromolecule contamination. A dielectric pad was placed underneath the occipital lobe to increase B1 efficiency and homogeneity in the regions where the MRS voxels were placed (*76*). First and second order shims were adjusted for the MRS voxel using FASTMAP (fast, automatic shimming technique by mapping along projections) with echo-planar imaging readout (*81*). During MRS data acquisition, participants were asked to complete the orientation discrimination task at the trained orientation for a total of 288 trials. No feedback was given to the participant.

Behavioral data analysis

Performance was measured by the 3-down-1-up staircase with 15 reversals. The mean angle difference of the last 8 reversals was taken as the threshold of each staircase run. The measured orientation discrimination thresholds were used as the dependent factor. Using a within-subject factorial design, we manipulated two independent factors, the reference orientation (trained and untrained orientation) and test session (pre-training, post-training), to evaluate the learning effect and the learning specificity. Further, we calculated the mean percent improvement (MPI) index for each condition: $MPI_{behaviour} = \frac{Threshold_{Pre} - Threshold_{post}}{Threshold_{Pre}} \times 100\%$. For statistical analysis, we used repeated-measures ANOVAs to compare across conditions.

MRI data analysis

Anatomical data analyses. T1-weighted anatomical data were used for coregistration and 3D cortex reconstruction. As a scan was acquired for each of the two sessions, the one with the best quality was selected to be used. Grey and white matter segmentation was obtained on the MP2RAGE images using FreeSurfer (https://surfer.nmr.mgh.harvard.edu/) and manually improved for the region-of-interest (right V1) using ITK-SNAP (http://www.itksnap.org). The refined segmentation was used to obtain a measurement of cortical thickness. Following previous studies, we assigned voxels to three layers (superficial, middle, deeper) using the equi-volume approach (*82*, *83*) as implemented in BrainVoyager (Brain Innovation, Maastricht, The Netherlands). This approach has been shown to reduce misclassification of voxels to layers, in particular for regions of interest presenting high curvature. Information from the cortical thickness map and gradient curvature was used to generate four grids at different cortical depths (ranging from 0: white matter, to 1: grey matter). Mapping of each voxel to a layer was obtained by

computing the Euclidean distance of each grey matter voxel to the grids: the two closest grids represent the borders of the layer to which a voxel is assigned. The anatomical image was aligned to the functional data using the boundary-based registration (*84*). We assessed the alignment and manually corrected if necessary. In particular, we checked: (1) the segmentation of the anatomical data; (2) the distortion correction of the functional data; (3) whether truncating the functional data improved the registration (*21*).

Functional MRI data analyses. Following previous work (*21*), the GE-EPI data were preprocessed using BrainVoyager (version 20.6, Brain Innovation, Maastricht, The Netherlands) and analysed using custom MATLAB (The MATHWORKS Inc., Natick, MA, USA) code. The first four volumes at the beginning of each run were discarded to ensure that the longitudinal magnetization reached steady state. Preprocessing of the functional data involved three steps starting with correction of distortions due to non-zero off-resonance field; that is, at the beginning of each functional run, five volumes with inverted phase encoding direction were acquired and used to estimate a voxel displacement map that was subsequently applied to the functional data using COPE (Correction based on Opposite Phase Encoding, BrainVoyager, Brain Innovation). The distortion-corrected data underwent slice-timing correction, head motion correction (the single-band image acquired at the beginning of the run closest in time to the acquisition of the anatomical scan, was used as the reference in the alignment), high-pass temporal filtering (using a GLM with Fourier basis set at 2 cycles) and removal of linear trends.

Regions of Interest definition. A binary mask was obtained for each participant based on anatomical templates provided by Benson (*85*) to define a region-of-interest (ROI) of right V1. This procedure used the individual participant-based segmentation obtained with FreeSurfer and an anatomical probabilistic template, to estimate the best location for the ROI. Each ROI was

subsequently inspected to ensure consistent definition across participants; that is, we checked whether the ROI (1) was located at the calcarine sulcus, (2) covered only gray matter, rather than white matter, or CSF.

We then modelled BOLD signals using a GLM with two regressors (i.e., stimulus vs. fixation) and included the estimated head motion parameters as nuisance regressors. The resulting t-statistical map was thresholded (t = 1.96, $p = 0.05$) to select voxels that responded strongly to the stimulus presentation.

Correcting for vasculature-related effects. Voxel selection within the right V1 was further refined by excluding voxels that were confounded by vasculature effects that are known to contribute to a superficial bias in the measured BOLD signal; that is, increased BOLD with increasing distance from white matter (*21*). In particular, it has been shown that the BOLD signal measured using GE-EPI (i.e., T2* weighted) is confounded by macro- and micro-vasculature signals (*86–88*). The macro-vasculature contribution is due to veins penetrating the grey matter and running through its thickness, as well as large pial veins situated along the surface of the grey matter (*89*). This results in increased sensitivity (i.e., strong BOLD effect) but decreased spatial specificity of the measured signal. The latter can be understood by the mechanics of the draining veins carrying deoxygenated haemoglobin downstream from the true neuronal site of neural activation, leading to a response spatially biased towards the pial surface, an effect known as superficial bias.

Here, we took the following approach to reduce superficial bias due to vasculature contributions. First, following previous work (*90–92*), we computed the temporal signal to noise ratio (tSNR) for each voxel in the ROI (i.e., right V1). We used tSNR to identify voxels near large veins that are expected to have large variance and low intensity signal due to the local

concentration of deoxygenated haemoglobin resulting in a short T2* decay time (i.e., dark intensity in a T2* weighted image). We identified voxels with low tSNR, checked their correspondence with voxels of lower intensities on the T2* weighted images. Second, it has been shown that high t-values on a fMRI statistical map are likely to arise from large pial veins (*93*, *94*). Therefore, voxels with low tSNR values or t-score values above the 90th percentile of the t-score distribution obtained by the GLM described above were removed from further analysis. We used these two approaches to correct the BOLD signal from confounding vasculature effects.

Further, following previous work (*21*), a spatial regression approach was used to control for signal contribution from draining veins. In particular, the intra-cortical veins running perpendicular to the cortical surface are known to drain blood from deeper layers of the cortex to larger pial veins situated along the gray matter surface, resulting in loss of spatial specificity and intra-layer BOLD signal contamination. To unmix the signals from adjacent layers, for each voxel in the superficial layer, we identified the nearest neighboring voxels in the middle layers. We then regressed out the mean time course of these voxels assigned to middle layers from the time course of these voxels assigned to superficial layers.

Univariate analysis. For each participant, test session, run and condition, we extracted the z-scored fMRI responses between the 4th and 9th TR (i.e., 6.18 – 16.48 s) after block onset. This time window captured the peak of the hemodynamic responses to the visual stimuli. The normalized fMRI responses were averaged across time points, blocks and runs for each condition and each session. Repeated-measures ANOVA was used to test the univariate difference across conditions. Comparing mean normalized fMRI responses across orientations and sessions did not show any significant results (two-way interaction [orientation × session] in the superficial layers: $F(2,54) = 1.493$, $p = 0.234$; middle layers: $F(2,54) = 1.252$, $p = 0.294$; and deeper layers: $F(2,54) = 2.236$, p

= 0.117), suggesting that the learning-dependent effects we observed reflect changes in orientation-specific representations across voxel patterns rather than mean univariate fMRI responses.

Multivariate pattern analysis. We used multivariate pattern analysis (MVPA) to decode: a) trained vs. control orientation, b) untrained vs. control orientation. Within the ROI for each participant, we calculated per voxel a t-score statistic by comparing activity for stimulus vs. fixation. We used this statistic to rank the voxels within the ROI and selected 300 voxels with the higher t-score to include in the MVPA, as classification accuracy saturated across all participants for these voxel pattern sizes in the corresponding regions. This voxel selection procedure ensured that comparisons of MVPA accuracy could not be confounded by varying number of voxels across participants. We then extracted mean normalized fMRI responses between 4th to 9th TR (i.e., 6.18 – 16.48 s) after block onset for this pattern of voxels per ROI, participant and test session. We trained a linear classifier using LIBSVM (http://www.csie.ntu.edu.tw/~cjlin/libsvm/) implemented in MATLAB to discriminate: a) the trained from the control orientation, b) the untrained from the control orientation. As both the trained and untrained orientation differed equally from the control orientation (~55°), we hypothesized that differences in the accuracy between these two classification tasks would be due to training rather than stimulus differences. We computed classification accuracy using blockwise cross-validation. That is, we divided the data set into training and test set—66, 54, or 42 training patterns (for 8 participants with 6 runs, 17 participants with 5 runs, and 3 participants with 4 runs, respectively), and 6 test patterns. We averaged the classification accuracy across folds, separately for each test session. We used repeated-measures ANOVAs and permutation tests to assess differences in classification accuracy across conditions (orientation × session). One participant was excluded due to lack of enough EPI data for the MPVA.

Mahalanobis distance analysis. To test whether training enhances the representation distance among different orientations or reduces the representation variance across different blocks, we implemented a Mahalanobis distance analysis. In particular, we extracted the mean normalized fMRI responses between 4th to 9th TR (i.e., 6.18 – 16.48 s) after block onset for 300 voxels per ROI, participant and test session. We then used principal component analysis (PCA) to select the most discriminative features, reducing data dimensionality from 300 (voxels) to 15 (principal components: PCs). The PC number was selected to ensure that the data following PCA explain 70% of the variance of the raw data (*95*). We calculated the within-condition and across condition distances using the Matlab (MathWorks) function 'mahal'. In particular, for each participant and each orientation (i.e., trained, untrained, control), we have M points (i.e., M blocks for each orientation) in the 15-dimensional space. For each data point in the trained (or untrained) orientation, we computed its distance to the control distribution (i.e., between-orientation distance) and to the trained (or untrained) orientation distribution (i.e., with-orientation distance). We next calculated the mean value for the between-orientation distance to estimate the representation distance between distributions (e.g., trained to control orientation). Note that this representation distance considers only the variability of the control orientation distribution. In addition, we calculated the mean of the within-orientation distance to estimate the representation variance within each distribution (i.e., block-wise variability within each orientation). Next, for each data point in the trained (or untrained) orientation, we calculated the ratio of between-orientation distance to within-orientation distance. A ratio larger than 1 indicates that a given data point is closer to the trained (or untrained) orientation distribution compared to the control orientation distribution. Therefore, the representation distance/variance provides a robust quantitative measure of stimulus

discriminability. We used repeated-measures ANOVAs and permutation tests to assess differences in representation distances across conditions (orientation × session).

Permutation tests. We conducted permutation tests by randomly shuffling the values for each participant for 1000 times. F-statistics or t-statistics were then computed on the shuffled data, creating a null distribution of F-statistics or t-statistics. The final p-values for F-statistics were determined based on the proportion of iterations where the shuffled F-statistics was equal to or greater than the actual F-statistics. The final p-values for t-statistics were determined based on the proportion of iterations where the shuffled t-statistics was equal to or greater than the actual t-statistics, and the proportion of iterations where the actual t-statistics was equal to or greater than the shuffled t-statistics, taking the minimum and multiplying by 2 (*54*).

MRS data analyses

MRS data were pre-processed with Eddy Current Correction (ECC2 + zero phase), phase (least-square option) and frequency (cross-correlation option) correction using MRspa v1.5 (https://www.cmrr.umn.edu/downloads/mrspa/). LCModel (*96*) was used to quantify standard metabolite concentrations in the range of 0.5 to 4.2 ppm using custom-chosen optimal initialization parameters.

Following previous work (*30*, *39*), the model spectra of alanine (Ala), aspartate (Asp), ascorbate/vitamin C (Asc), glycerophosphocholine (GPC), phosphocholine (PCho), creatine (Cr), phosphocreatine (PCr), γ-aminobutyric acid (GABA), glucose (Glc), glutamine (Gln), Glutamate (Glu), glutathione (GSH), myo-inositol (Ins), N-acetylaspartate (NAA), N-acetylaspartylglutamate (NAAG), phosphoethanolamine (PE), scyllo-inositol (Scyllo) and taurine (Tau) were generated based on previously reported chemical shifts and coupling constants by using

GAMMA/PyGAMMA simulation library of VESPA (Versatile Simulation, Pulses and Analysis) for carrying out the density matrix formalism. Simulations were performed with the same RF pulses and sequence timings as used in the study. We followed the same macromolecule inclusion procedure as Bednařík et al (*97*). Macromolecule spectra acquired from the occipital cortex from 3 healthy volunteers, using an inversion recovery sequence (TR=3 s, TE=36 ms, inversion time TI=0.685 s), were included in the LCModel basis set. The residual signal of the methylene of tCr at 3.93 ppm was removed by post processing and the high-frequency noise was suppressed using a Gaussian filter (σ=0.05 s) before including the macromolecule spectrum into the LCModel basis set.

We referenced metabolite concentrations to the sum of the concentrations of Creatine (Cr) and Phosphocreatine (PCr), that is total Creatine (tCr). We chose tCr as reference for two main reasons. First, tCr concentration was measured in the same spectrum, concurrently with GABA, while water concentration was estimated from a different scan. Using another metabolite acquired in the same spectrum as reference accounts for the possibility of small but relevant changes in neuronal density and spectral data quality that might be expected during periods of task activity. Second, referencing metabolites to tCr has been shown to have better reproducibility compared to other referencing methods (*98*) and has been widely used as a reference metabolite in MRS studies (*99*, *100*). Our control analysis confirmed that tCr concentration did not change significantly during training across sessions, suggesting that our results are specific to GABA changes and are not driven by changes in tCr concentration. To ensure results were not driven by the chosen reference, we replicated our findings referencing GABA and Glu concentrations to water (*39*, *101*, *102*).

All MRspa spectral linewidths were below 15.5 Hz (i.e., two standard deviations from the mean across participants) and GABA Cramer-Rao-Lower-Bound (CRLB) values were less than 20% with no visible lipid contamination (determined by visual inspection by two independent reviewers, JZ, CS). Four participants were excluded from MRS analyses, after controlling for data quality (either GABA CRLB > 20 % or MRspa linewidths > 15.50 Hz). The voxel percentage of grey matter (*fGM*), white matter (*fWM*) and cerebrospinal fluid (*fCSF*) in the MRS voxel was calculated from the segmented MP2RAGE maps. Correction for voxel tissue composition was performed by 1) accounting for GM voxel fraction with the α-correction method, 2) dividing GABA and Glu concentration by 1 – fCSF (fraction of CSF in the MRS voxel).

In particular, for alpha correction we used the approach described by Harris et al (*103*); this method assumes that the concentration of GABA in the grey matter is double that of white matter, and it is summarized by the following formula:

$$c\text{GMWMcorr} = \frac{c}{fGM + \alpha fWM} + \frac{\mu GM + \alpha \mu WM}{\mu GM + \mu WM}$$

Where C represents the concentration of GABA output by LCModel, fGM and fWM represent the fraction of gray and white matter (respectively) in the voxel of interest, and the alpha value is assumed to be 0.5 (*103*). μGM and μWM are the GM and WM fractions of the group average voxel fractions across participants, and are driven by fractions in each specific dataset.

1-CSF correction makes no assumptions about the distribution of GABA or Glutamate between grey and white matter and divides neuro-metabolites concentration by 1-CSF (*104*).

$$c\text{CSFcorr} = \frac{C}{fGM + fWM}$$

We calculated the ratio between GABA and Glu concentration as an index of inhibition/excitation in early visual cortex. We also obtained a MPI index for this ratio in the form of $MPI_{GABA/Glu} = \frac{GABA/Glu_{post} - GABA/Glu_{pre}}{GABA/Glu_{pre}} \times 100\%$.

Note that limitations in MRS spatial resolution result in differences between the MRS voxel size and fMRI coverage of V1. We calculated the percentage of overlap between the MRS voxel and the right V1 ROI used for fMRI analyses, as defined by anatomical probabilistic template. The mean overlap across participants was 44.3% for session 1 (SD = 8.0%) and 43.4% for session 2 (SD = 8.0%). We also confirmed that the MRS voxel had minimal overlap with V2 (session 1: mean = 7.1%, SD = 5.7%; session 2: mean = 6.8%, SD = 5.0%), V3 (session 1: mean = 1.4%, SD = 1.9%; session 2: mean = 1.4%, SD = 1.8%), and V4 (session 1: mean = 0%, SD = 0%; session 2: mean = 0.003%, SD = 0.015%) (all right hemisphere, masks obtained from Wang et al., 2015). For each participant, we used this overlap between the MRS voxel and V1 ROI to normalize GABA and Glu concentrations (i.e., divided GABA and Glu concentrations by the overlap) for further analyses (mean differences across sessions, correlations with behavior, fMRI) to account for differences in their overlap across participants.

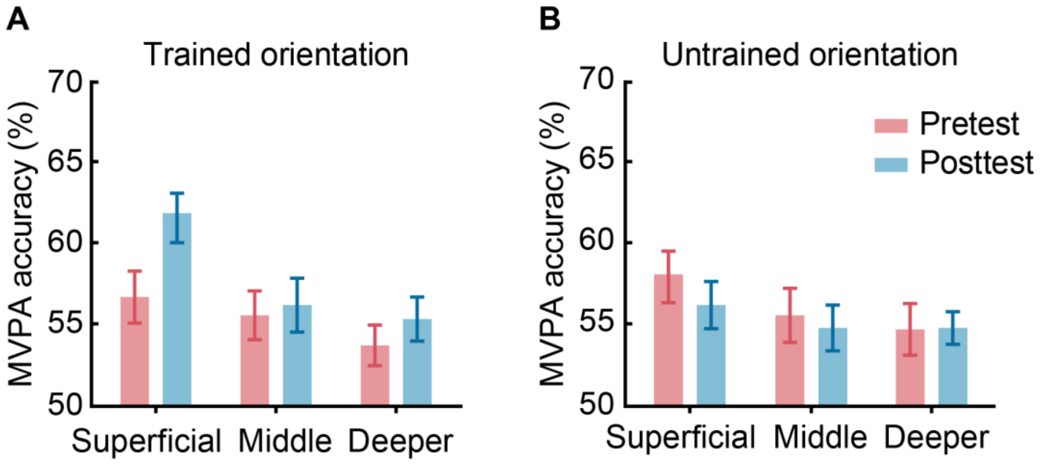

Fig. S1. **Replication of learning-dependent changes in orientation-specific representations in superficial V1 layers.** Our fMRI results provide an independent replication of the layer-specific learning effect (i.e., increased MVPA accuracy for the trained orientation) we reported in our previous work (*21*). Pooling data across two independent participant samples (i.e., including the data from (*21*)) showed a significant three-way interaction (orientation × session × layer) on MVPA accuracy ($F(2,80) = 3.503$, $p = 0.035$, permutation test: $p = 0.037$). Further two-way repeated-measures ANOVA showed a session × orientation interaction in the superficial ($F(1,40) = 14.699$, $p < 0.001$, permutation test: $p < 0.001$), rather than middle ($F(1,40) = 0.577$, $p = 0.452$, permutation test: $p = 0.454$) or deeper ($F(1,40) = 0.516$, $p = 0.477$, permutation test: $p = 0.482$) V1 layers.

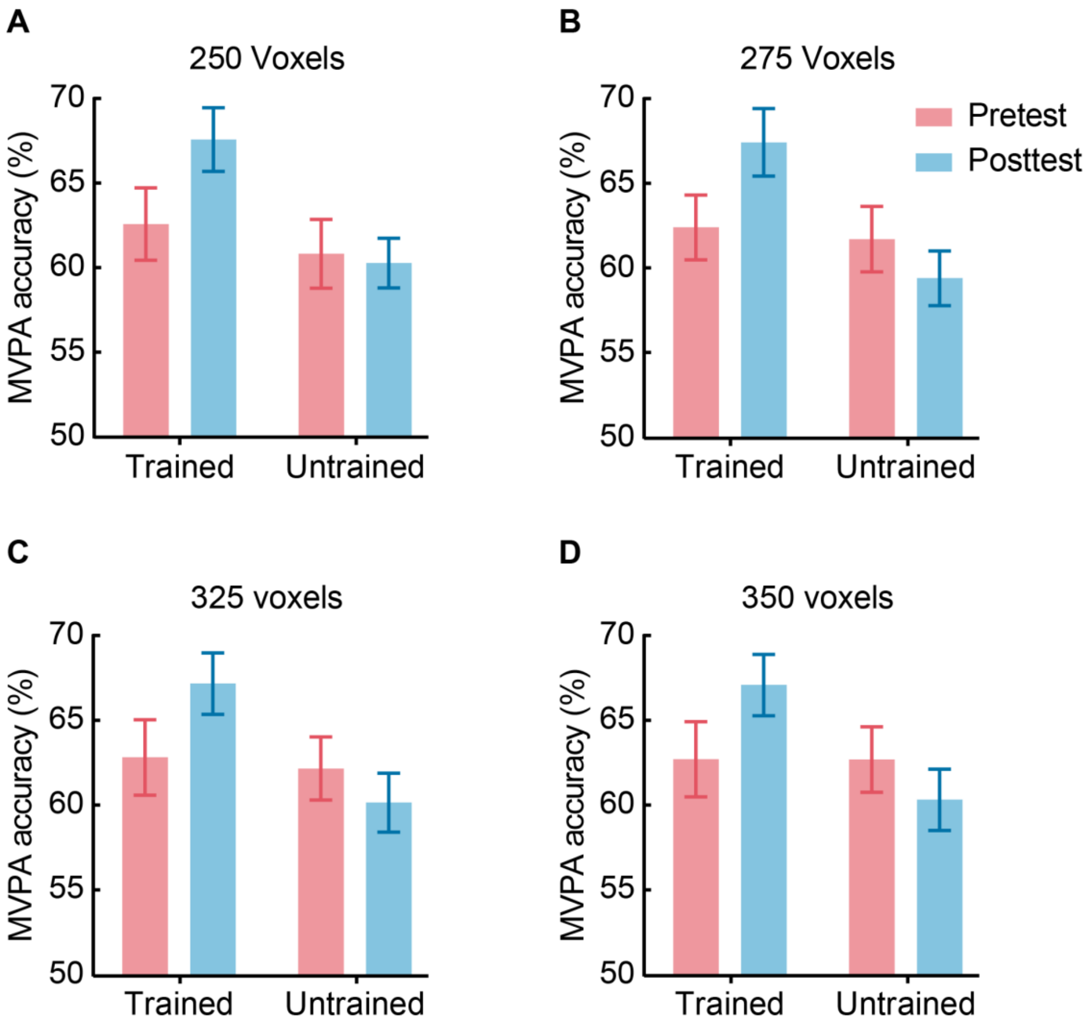

Fig. S2. Learning-dependent changes in superficial V1 layers for the trained orientation across MVPA pattern sizes. We observed similar learning-dependent changes in MVPA accuracy in superficial V1 layers for the trained orientation across different pattern sizes: 250 voxels ($F(1,27) = 4.819$, $p = 0.037$, permutation test: $p = 0.033$), 275 voxels ($F(1,27) = 8.504$, $p = 0.007$, permutation test: $p = 0.003$), 325 voxels ($F(1,27) = 6.353$, $p = 0.018$, permutation test: $p = 0.020$), and 350 voxels ($F(1,27) = 7.359$, $p = 0.011$, permutation test: $p = 0.008$).

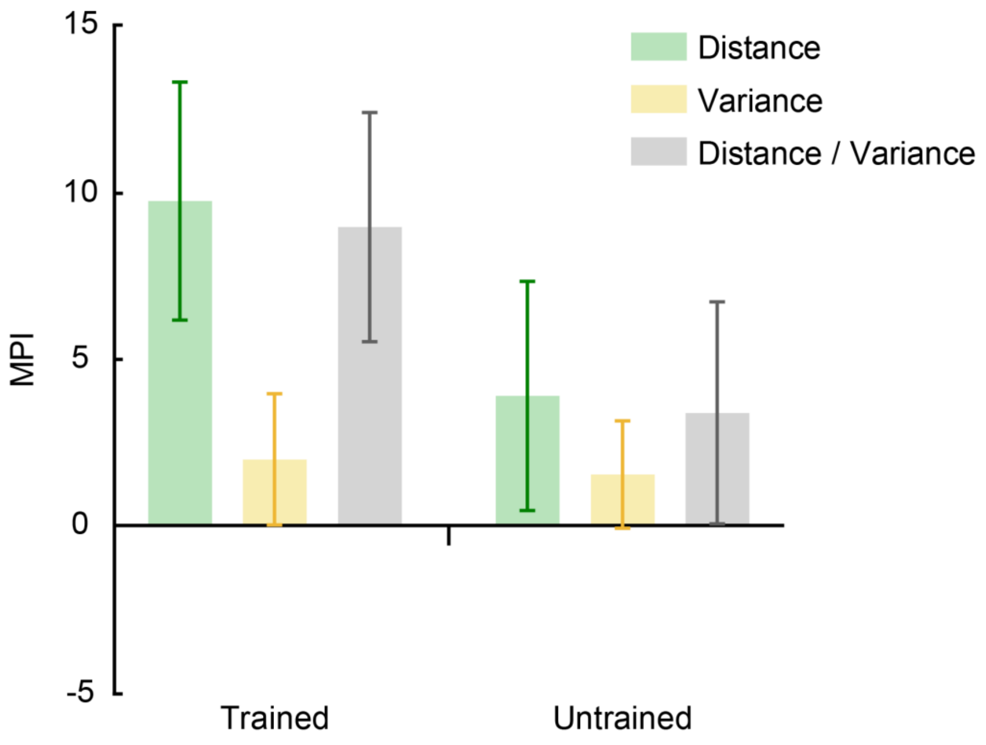

Fig. S3. MPI for representation distance, variance and distance/variance.

Mean improvement index (MPI) showed significant changes in representation distance and distance/variance rather than variance for the trained compared to the untrained orientation.

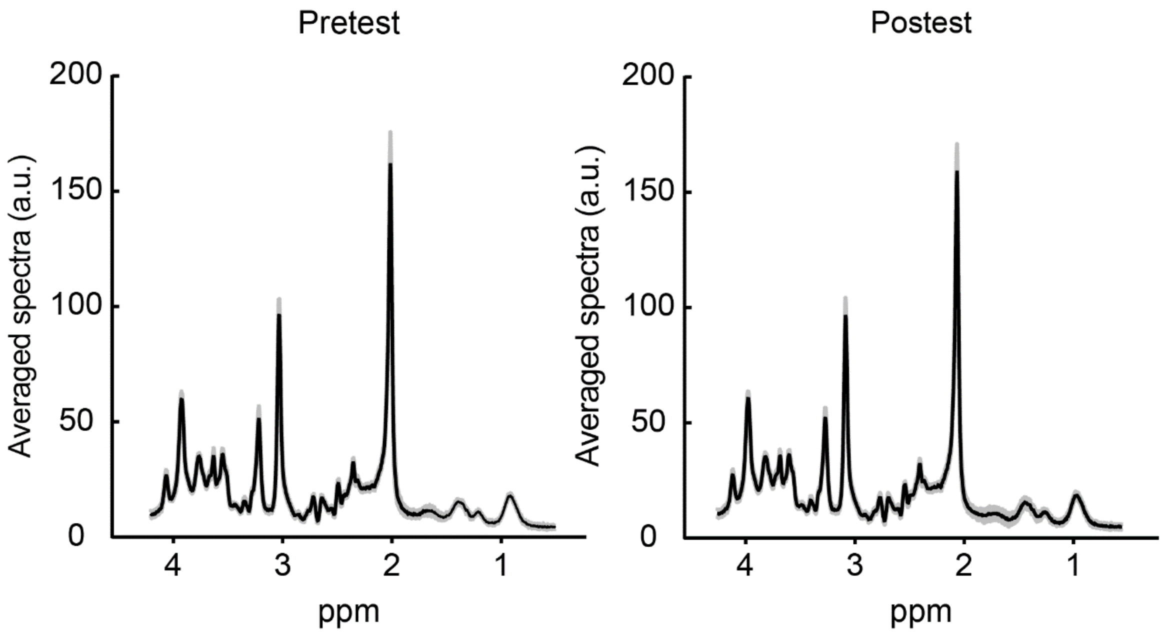

Fig. S4. Average MRS spectra across participants for pretest and posttest sessions.

Mean (solid black line) ± standard deviation (shaded) of averaged spectra (arbitrary unit, a.u.) from early visual cortex across participants.

MRS Measures	Scan	Mean	Standard Deviation
Linewidth	Pre-training	0.032	0.004
	Post-training	0.033	0.003
SNR	Pre-training	63.600	7.810
	Post-training	64.120	7.305
tCr	Pre-training	17.536	3.499
	Post-training	17.483	3.574
Overlap with right V1	Pre-training	0.443	0.080
	Post-training	0.434	0.080

Table S1. MRS quality measures (mean and standard deviation per scanning session). There were no significant differences in data quality measures across sessions (paired t-test, Linewidth: $t(24) = -0.699$, $p = 0.491$; SNR: $t(24) = -0.446$, $p = 0.659$; overlap with right V1: $t(24) = 0.798$, $p = 0.433$; concentration of tCr: $t(24) = 0.223$, $p = 0.826$, normalised to the overlap between the MRS voxel and the V1 ROI).

Table S2. Minimum Reporting Standards in MRS checklist

Site (Name or Number)	WBIC – Wolfson Brain Imaging Centre (University of Cambridge)
1. Hardware	
a. Field strength [T]	7
b. Manufacturer	Siemens
c. Model	Terra
d. RF coils	one-transmit coil with integrated 32 receive element head coil
e. Additional hardware	Dielectric pads
2. Acquisition	
a. Pulse sequence	Semi-LASER
b. Volume of Interest (VOI) locations	Right Occipital Cortex (EV)
c. Nominal VOI size [mm^3]	15x15x15 mm^3
d. Repetition Time (TR), Echo Time (TE)	TR=5000ms, TE=36ms
e. Total number of Excitations or acquisitions per spectrum	128
f. Additional sequence parameters:	Spectral bandwidth: 6000 Hz<br>Spectral points: 2048
g. Water Suppression Method	VAPOR
h. Shimming Method, reference peak, and thresholds for "acceptance of shim" chosen	Automated 3D head shim (GRE - BRAIN) and second order shim adjusted using FASTMAP to achieve water peak linewidth below 15 Hz
i. Triggering or motion correction method	N/A
3. Data analysis methods and outputs	
a. Analysis software	MRspa (preprocessing, version v1.5c), LCModel (fitting and quantification)
b. Processing steps deviating from quoted reference or product	MRspa pre-processing options selected:<br>- eddy current corr.: ECC2 + zero phase<br>- frequency corr.: absolute (3.01)<br>- phase corr.: least square
c. Output measure	Tissue-corrected concentrations relative to water or tCr

d. Quantification references and assumptions, fitting model assumptions	The model spectra of alanine (Ala), aspartate (Asp), ascorbate/vitamin C (Asc), glycerophosphocholine (GPC), phosphocholine (PCho), creatine (Cr), phosphocreatine (PCr), γ-amino-butyric acid (GABA), glucose (Glc), glutamine (Gln), Glutamate (Glu), glutathione (GSH), myo-inositol (Ins), Nacetylaspartate (NAA), Nacetylaspartylglutamate (NAAG), phosphoethanolamine (PE), scyllo-inositol (Scyllo) and taurine (Tau) were generated based on previously reported chemical shifts and coupling constants by using GAMMA/PyGAMMA simulation library of VESPA (Versatile Simulation, Pulses and Analysis) for carrying out the density matrix formalism.
4. Data Quality	
a. Reported variables	See Table S1
b. Data exclusion criteria	Water peak linewidth > 15.5 Hz<br>CRLB > 20 %
c. Quality measures of postprocessing Model fitting	See Table S1
d. Sample Spectrum	See Figure S4

REFERENCES AND NOTES

1. K. P. Berry, E. Nedivi, Experience-dependent structural plasticity in the visual system. *Annu. Rev. Vis. Sci.* **2**, 17–35 (2016).

2. C. D. Gilbert, W. Li, Adult visual cortical plasticity. *Neuron* **75**, 250–264 (2012).

3. B. Dosher, Z.-L. Lu, Visual perceptual learning and models. *Annu. Rev. Vis. Sci.* **3**, 343–363 (2017).

4. C. T. Law, J. I. Gold, Shared mechanisms of perceptual learning and decision making. *Top. Cogn. Sci.* **2**, 226–238 (2010).

5. Z. Kourtzi, J. J. DiCarlo, Learning and neural plasticity in visual object recognition. *Curr. Opin. Neurobiol.* **16**, 152–158 (2006).

6. D. Sagi, Perceptual learning in vision research. *Vision Res.* **51**, 1552–1566 (2011).

7. R. W. Li, D. M. Levi, S. A. Klein, Perceptual learning improves efficiency by re-tuning the decision 'template' for position discrimination. *Nat. Neurosci.* **7**, 178–183 (2004).

8. S.-G. Kuai, D. Levi, Z. Kourtzi, Learning optimizes decision templates in the human visual cortex. *Curr. Biol.* **23**, 1799–1804 (2013).

9. K. Jia, P. Frangou, V. M. Karlaftis, J. J. Ziminski, J. Giorgio, R. Rideaux, E. Zamboni, V. Hodgson, U. Emir, Z. Kourtzi, Neurochemical and functional interactions for improved perceptual decisions through training. *J. Neurophysiol.* **127**, 900–912 (2022).

10. A. F. Teich, N. Qian, Learning and adaptation in a recurrent model of V1 orientation selectivity. *J. Neurophysiol.* **89**, 2086–2100 (2003).

11. L. Schwabe, K. Obermayer, Adaptivity of tuning functions in a generic recurrent network model of a cortical hypercolumn. *J. Neurosci.* **25**, 3323–3332 (2005).

12. J. Goense, Y. Bohraus, N. K. Logothetis, fMRI at high spatial resolution: Implications for BOLD-models. *Front. Comput. Neurosci.* **10**, 66 (2016).

13. J. Yang, L. Huber, Y. Yu, P. A. Bandettini, Linking cortical circuit models to human cognition with laminar fMRI. *Neurosci. Biobehav. Rev.* **128**, 467–478 (2021).

14. K. Jia, R. Goebel, Z. Kourtzi, Ultra-high field imaging of human visual cognition. *Annu. Rev. Vis. Sci.* **9**, 479–500 (2023).

15. M. W. Self, T. van Kerkoerle, R. Goebel, P. R. Roelfsema, Benchmarking laminar fMRI: Neuronal spiking and synaptic activity during top-down and bottom-up processing in the different layers of cortex. *Neuroimage* **197**, 806–817 (2019).

16. S. J. D. Lawrence, E. Formisano, L. Muckli, F. P. de Lange, Laminar fMRI: Applications for cognitive neuroscience. *Neuroimage* **197**, 785–791 (2019).

17. J. F. M. Jehee, S. Ling, J. D. Swisher, R. S. Van Bergen, F. Tong, Perceptual learning selectively refines orientation representations in early visual cortex. *J. Neurosci.* **32**, 16747–16753 (2012).

18. A. Schoups, R. Vogels, N. Qian, G. Orban, Practising orientation identification improves orientation coding in V1 neurons. *Nature* **412**, 549–553 (2001).

19. L. Q. Xiao, J. Y. Zhang, R. Wang, S. A. Klein, D. M. Levi, C. Yu, Complete transfer of perceptual learning across retinal locations enabled by double training. *Curr. Biol.* **18**, 1922–1926 (2008).

20. Y. Z. Xiong, J. Y. Zhang, C. Yu, Bottom-up and top-down influences at untrained conditions determine perceptual learning specificity and transfer. *eLife* **5**, e14614 (2016).

21. K. Jia, E. Zamboni, V. Kemper, C. Rua, N. R. Goncalves, A. K. T. Ng, C. T. Rodgers, G. Williams, R. Goebel, Z. Kourtzi, Recurrent processing drives perceptual plasticity. *Curr. Biol.* **30**, 4177–4187.e4 (2020).

22. J. Gold, P. J. Bennett, A. B. Sekuler, Signal but not noise changes with perceptual learning. *Nature* **402**, 176–178 (1999).

23. M. L. Caras, D. H. Sanes, Top-down modulation of sensory cortex gates perceptual learning. *Proc. Natl. Acad. Sci. U.S.A.* **114**, 9972–9977 (2017).

24. Y. Yan, M. J. Rasch, M. Chen, X. Xiang, M. Huang, S. Wu, W. Li, Perceptual training continuously refines neuronal population codes in primary visual cortex. *Nat. Neurosci.* **17**, 1380–1387 (2014).

25. H. Z. Adab, R. Vogels, Practicing coarse orientation discrimination improves orientation signals in macaque cortical area V4. *Curr. Biol.* **21**, 1661–1666 (2011).

26. D. Ferster, K. D. Miller, Neural mechanisms of orientation selectivity in the visual cortex. *Annu. Rev. Neurosci.* **23**, 441–471 (2000).

H. Yu, X. Chen, C. Sun, T. Shou, Global evaluation of contributions of $GABA_A$, AMPA and NMDA receptors to orientation maps in cat's visual cortex. *Neuroimage* **40**, 776–787 (2008).

28. K. Shibata, Y. Sasaki, J. W. Bang, E. G. Walsh, M. G. Machizawa, M. Tamaki, L.-H. Chang, T. Watanabe, Overlearning hyperstabilizes a skill by rapidly making neurochemical processing inhibitory-dominant. *Nat. Neurosci.* **20**, 470–475 (2017).

29. J. W. Bang, K. Shibata, S. M. Frank, E. G. Walsh, M. W. Greenlee, T. Watanabe, Y. Sasaki, Consolidation and reconsolidation share behavioural and neurochemical mechanisms. *Nat. Hum. Behav.* **2**, 507–513 (2018).

30. P. Frangou, U. E. Emir, V. M. Karlaftis, C. Nettekoven, E. L. Hinson, S. Larcombe, H. Bridge, C. J. Stagg, Z. Kourtzi, Learning to optimize perceptual decisions through suppressive interactions in the human brain. *Nat. Commun.* **10**, 474 (2019).

31. J. J. Ziminski, P. Frangou, V. M. Karlaftis, U. Emir, Z. Kourtzi, Microstructural and neurochemical plasticity mechanisms interact to enhance human perceptual decision-making. *PLOS Biol.* **21**, e3002029 (2023).

32. J. Zhang, A. Meeson, A. E. Welchman, Z. Kourtzi, Learning alters the tuning of functional magnetic resonance imaging patterns for visual forms. *J. Neurosci.* **30**, 14127–14133 (2010).

33. J. Dobres, A. R. Seitz, Perceptual learning of oriented gratings as revealed by classification images. *J. Vis.* **10**, 8 (2010).

34. N. Dupuis-Roy, F. Gosselin, Perceptual learning without signal. *Vision Res.* **47**, 349–356 (2007).

35. C.-T. Law, J. I. Gold, Neural correlates of perceptual learning in a sensory-motor, but not a sensory, cortical area. *Nat. Neurosci.* **11**, 505–513 (2008).

36. R. A. E. Edden, S. D. Muthukumaraswamy, T. C. A. Freeman, K. D. Singh, Orientation discrimination performance is predicted by GABA concentration and gamma oscillation frequency in human primary visual cortex. *J. Neurosci.* **29**, 15721–15726 (2009).

37. P. Frangou, M. Correia, Z. Kourtzi, GABA, not BOLD, reveals dissociable learning-dependent plasticity mechanisms in the human brain. *eLife* **7**, e35854 (2018).

38. I. B. Ip, H. Bridge, Investigating the neurochemistry of the human visual system using magnetic resonance spectroscopy. *Brain Struct. Funct.* **227**, 1491–1505 (2022).

39. C. Lunghi, U. E. Emir, M. C. Morrone, H. Bridge, Short-term monocular deprivation alters GABA in the adult human visual cortex. *Curr. Biol.* **25**, 1496–1501 (2015).

40. C. J. Stagg, V. Bachtiar, H. Johansen-Berg, The role of GABA in human motor learning. *Curr. Biol.* **21**, 480–484 (2011).

41. T. Yamada, T. Watanabe, Y. Sasaki, Plasticity–stability dynamics during post-training processing of learning. *Trends Cogn. Sci.* **28**, 72–83 (2023).

42. I. B. Ip, U. E. Emir, A. J. Parker, J. Campbell, H. Bridge, Comparison of neurochemical and BOLD signal contrast response functions in the human visual cortex. *J. Neurosci.* **39**, 7968–7975 (2019).

43. R. S. Koolschijn, A. Shpektor, W. T. Clarke, I. Betina Ip, D. Dupret, U. E. Emir, H. C. Barron, Memory recall involves a transient break in excitatory-inhibitory balance. *eLife* **10**, e70071 (2021).

44. M. Wehr, A. M. Zador, Balanced inhibition underlies tuning and sharpens spike timing in auditory cortex. *Nature* **426**, 442–446 (2003).

45. K. M. Hagihara, K. Ohki, Long-term down-regulation of GABA decreases orientation selectivity without affecting direction selectivity in mouse primary visual cortex. *Front. Neural Circuits* **7**, 28 (2013).

46. G. Li, Y. Yang, Z. Liang, J. Xia, Y. Yang, Y. Zhou, GABA-mediated inhibition correlates with orientation selectivity in primary visual cortex of cat. *Neuroscience* **155**, 914–922 (2008).

47. S. Shushruth, P. Mangapathy, J. M. Ichida, P. C. Bressloff, L. Schwabe, A. Angelucci, Strong recurrent networks compute the orientation tuning of surround modulation in the primate primary visual cortex. *J. Neurosci.* **32**, 308–321 (2012).

48. R. J. Douglas, K. A. C. Martin, Recurrent neuronal circuits in the neocortex. *Curr. Biol.* **17**, R496–R500 (2007).

49. C. D. Gilbert, T. Wiesel, Clustered intrinsic connections in cat visual cortex. *J. Neurosci.* **3**, 1116–1133 (1983).

50. K. S. RockLand, D. N. Pandya, Laminar origins and terminations of cortical connections of the occipital lobe in the rhesus monkey. *Brain Res.* **179**, 3–20 (1979).

51. A. G. Khan, J. Poort, A. Chadwick, A. Blot, M. Sahani, T. D. Mrsic-flogel, S. B. Hofer, Distinct learning-induced changes in stimulus selectivity and interactions of GABAergic interneuron classes in visual cortex. *Nat. Neurosci.* **21**, 851–859 (2018).

52. J. Poort, K. A. Wilmes, A. Blot, A. Chadwick, M. Sahani, C. Clopath, T. D. Mrsic-flogel, S. B. Hofer, A. G. Khan, Learning and attention increase visual response selectivity through distinct mechanisms. *Neuron* **110**, 686–697.e6 (2022).

53. S. Moeller, E. Yacoub, C. A. Olman, E. Auerbach, J. Strupp, N. Harel, K. Uğurbil, Multiband multislice GE-EPI at 7 tesla, with 16-fold acceleration using partial parallel imaging with application to high spatial and temporal whole-brain FMRI. *Magn. Reson. Med.* **63**, 1144–1153 (2010).

54. R. L. Rademaker, C. Chunharas, J. T. Serences, Coexisting representations of sensory and mnemonic information in human visual cortex. *Nat. Neurosci.* **22**, 1336–1344 (2019).

55. J. Stelzer, Y. Chen, R. Turner, Statistical inference and multiple testing correction in classification-based multi-voxel pattern analysis (MVPA): Random permutations and cluster size control. *Neuroimage* **65**, 69–82 (2013).

56. C. R. Pernet, R. Wilcox, G. A. Rousselet, Robust correlation analyses: False positive and power validation using a new open source matlab toolbox. *Front. Psychol.* **3**, 606 (2013).

57. M. Dubol, C. N. Epperson, J. Sacher, B. Pletzer, B. Derntl, R. Lanzenberger, I. Sundström-Poromaa, E. Comasco, Neuroimaging the menstrual cycle: A multimodal systematic review. *Front. Neuroendocrinol.* **60**, 100878 (2021).

58. C. N. Epperson, S. O'Malley, K. A. Czarkowski, R. Gueorguieva, P. Jatlow, G. Sanacora, D. L. Rothman, J. H. Krystal, G. F. Mason, Sex, GABA, and nicotine: The impact of smoking on cortical GABA levels across the menstrual cycle as measured with proton magnetic resonance spectroscopy. *Biol. Psychiatry* **57**, 44–48 (2005).

59. C. Neill Epperson, K. Haga, G. F. Mason, E. Sellers, R. Gueorguieva, W. Zhang, E. Weiss, D. L. Rothman, J. H. Krystal, Cortical γ-aminobutyric acid levels across the menstrual cycle in healthy women and those with premenstrual dysphoric disorder: A proton magnetic resonance spectroscopy study. *Arch. Gen. Psychiatry* **59**, 851–858 (2002).

60. M. Harada, H. Kubo, A. Nose, H. Nishitani, T. Matsuda, Measurement of variation in the human cerebral GABA level by in vivo MEGA-editing proton MR spectroscopy using a clinical 3 T instrument and its dependence on brain region and the female menstrual cycle. *Hum. Brain Mapp.* **32**, 828–833 (2011).

61. T. De Bondt, F. De Belder, F. Vanhevel, Y. Jacquemyn, P. M. Parizel, Prefrontal GABA concentration changes in women—Influence of menstrual cycle phase, hormonal contraceptive use, and correlation with premenstrual symptoms. *Brain Res.* **1597**, 129–138 (2015).

62. B. Liu, G. Wang, D. Gao, F. Gao, B. Zhao, M. Qiao, H. Yang, Y. Yu, F. Ren, P. Yang, W. Chen, C. D. Rae, Alterations of GABA and glutamate-glutamine levels in premenstrual dysphoric disorder: A 3T proton magnetic resonance spectroscopy study. *Psychiatry Res.* **231**, 64–70 (2015).

63. J. Arrubla, D. H. Y. Tse, C. Amkreutz, I. Neuner, N. J. Shah, GABA concentration in posterior cingulate cortex predicts putamen response during resting state fMRI. *PLOS ONE* **9**, e106609 (2014).

64. T. Bell, E. S. Boudes, R. S. Loo, G. J. Barker, D. J. Lythgoe, R. A. E. Edden, R. M. Lebel, M. Wilson, A. D. Harris, In vivo Glx and Glu measurements from GABA-edited MRS at 3 T. *NMR Biomed.* **34**, e4245 (2021).

65. T. Bunai, T. Hirosawa, M. Kikuchi, M. Fukai, M. Yokokura, S. Ito, Y. Takata, T. Terada, Y. Ouchi, tDCS-induced modulation of GABA concentration and dopamine release in the human brain: A combination study of magnetic resonance spectroscopy and positron emission tomography. *Brain Stimul.* **14**, 154–160 (2021).

66. K. Heimrath, A. Brechmann, R. Blobel-Lüer, J. Stadler, E. Budinger, T. Zaehle, Transcranial direct current stimulation (tDCS) over the auditory cortex modulates GABA and glutamate: A 7 T MR-spectroscopy study. *Sci. Rep.* **10**, 20111 (2020).

67. L. F. Kaiser, T. O. J. Gruendler, O. Speck, L. Luettgau, G. Jocham, Dissociable roles of cortical excitation-inhibition balance during patch-leaving versus value-guided decisions. *Nat. Commun.* **12**, 904 (2021).

68. N. Levar, T. J. Van Doesum, D. Denys, G. A. Van Wingen, Anterior cingulate GABA and glutamate concentrations are associated with resting-state network connectivity. *Sci. Rep.* **9**, 2116 (2019).

69. M. Mikkelsen, A. D. Harris, R. A. E. Edden, N. A. J. Puts, Macromolecule-suppressed GABA measurements correlate more strongly with behavior than macromolecule-contaminated GABA+ measurements. *Brain Res.* **1701**, 204–211 (2018).

70. L. R. Silberbauer, B. Spurny, P. Handschuh, M. Klöbl, P. Bednarik, B. Reiter, V. Ritter, P. Trost, M. E. Konadu, M. Windpassinger, T. Stimpfl, W. Bogner, R. Lanzenberger, M. Spies, Effect of ketamine on limbic GABA and glutamate: A human in vivo multivoxel magnetic resonance spectroscopy study. *Front. Psych.* **11**, 549903 (2020).

71. A. Takacs, A. K. Stock, P. Kuntke, A. Werner, C. Beste, On the functional role of striatal and anterior cingulate GABA+ in stimulus-response binding. *Hum. Brain Mapp.* **42**, 1863–1878 (2021).

72. C. Barth, A. Villringer, J. Sacher, Sex hormones affect neurotransmitters and shape the adult female brain during hormonal transition periods. *Front. Neurosci.* **9**, 37 (2015).

73. D. G. Pelli, The VideoToolbox software for visual psychophysics: Transforming numbers into movies. *Spat. Vis.* **10**, 437–442 (1997).

74. D. H. Brainard, The psychophysics toolbox. *Spat. Vis.* **10**, 433–436 (1997).

75. G. Öz, I. Tkáč, Short-echo, single-shot, full-intensity proton magnetic resonance spectroscopy for neurochemical profiling at 4 T: Validation in the cerebellum and brainstem. *Magn. Reson. Med.* **65**, 901–910 (2011).

76. C. Lemke, A. Hess, S. Clare, V. Bachtiar, C. Stagg, P. Jezzard, U. Emir, Two-voxel spectroscopy with dynamic B0 shimming and flip angle adjustment at 7 T in the human motor cortex. *NMR Biomed.* **28**, 852–860 (2015).

77. M. Terpstra, I. Cheong, T. Lyu, D. K. Deelchand, U. E. Emir, P. Bednařík, L. E. Eberly, G. Öz, Test-retest reproducibility of neurochemical profiles with short-echo, single-voxel MR spectroscopy at 3T and 7T. *Magn. Reson. Med.* **76**, 1083–1091 (2016).

78. B. L. van de Bank, U. E. Emir, V. O. Boer, J. J. A. van Asten, M. C. Maas, J. P. Wijnen, H. E. Kan, G. Oz, D. W. J. Klomp, T. W. J. Scheenen, Multi-center reproducibility of neurochemical profiles in the human brain at 7 T. *NMR Biomed.* **28**, 306–316 (2015).

79. G. Zacharopoulos, F. Sella, U. Emir, R. Cohen Kadosh, Dissecting the chain of information processing and its interplay with neurochemicals and fluid intelligence across development. *eLife* **12**, e84086 (2023).

80. I. Tkáč, Z. Starčuk, I.-Y. Choi, R. Gruetter, In vivo ^{1}H NMR spectroscopy of rat brain at 1 ms echo time. *Magn. Reson. Med.* **41**, 649–656 (1999).

81. R. Gruetter, I. Tkáč, Field mapping without reference scan using asymmetric echo-planar techniques. *Magn. Reson. Med.* **43**, 319–323 (2000).

82. M. D. Waehnert, J. Dinse, M. Weiss, M. N. Streicher, P. Waehnert, S. Geyer, R. Turner, P. L. Bazin, Anatomically motivated modeling of cortical laminae. *Neuroimage* **93**, 210–220 (2014).

83. V. G. Kemper, F. De Martino, T. C. Emmerling, E. Yacoub, R. Goebel, High resolution data analysis strategies for mesoscale human functional MRI at 7 and 9.4 T. *Neuroimage* **164**, 48–58 (2018).

84. D. N. Greve, B. Fischl, Accurate and robust brain image alignment using boundary-based registration. *Neuroimage* **48**, 63–72 (2009).

85. L. Wang, R. E. B. Mruczek, M. J. Arcaro, S. Kastner, Probabilistic maps of visual topography in human cortex. *Cereb. Cortex* **25**, 3911–3931 (2015).

86. K. Uludağ, B. Müller-Bierl, K. Uğurbil, An integrative model for neuronal activity-induced signal changes for gradient and spin echo functional imaging. *Neuroimage* **48**, 150–165 (2009).

87. K. Uğurbil, L. Toth, D. S. Kim, How accurate is magnetic resonance imaging of brain function? *Trends Neurosci.* **26**, 108–114 (2003).

88. E. Yacoub, P. F. Van De Moortele, A. Shmuel, K. Uğurbil, Signal and noise characteristics of Hahn SE and GE BOLD fMRI at 7 T in humans. *Neuroimage* **24**, 738–750 (2005).

89. H. M. Duvernoy, S. Delon, J. L. Vannson, Cortical blood vessels of the human brain. *Brain Res. Bull.* **7**, 519–579 (1981).

90. A. K. T. Ng, K. Jia, N. R. Goncalves, E. Zamboni, V. G. Kemper, R. Goebel, A. E. Welchman, Z. Kourtzi, Ultra-high-field neuroimaging reveals fine-scale processing for 3d perception. *J. Neurosci.* **41**, 8362–8374 (2021).

91. K. Jia, E. Zamboni, C. Rua, N. R. Goncalves, V. Kemper, A. K. T. Ng, C. T. Rodgers, G. Williams, R. Goebel, Z. Kourtzi, A protocol for ultra-high field laminar fMRI in the human brain. *STAR Protoc.* **2**, 100415 (2021).

92. C. A. Olman, S. Inati, D. J. Heeger, The effect of large veins on spatial localization with GE BOLD at 3 T: Displacement, not blurring. *Neuroimage* **34**, 1126–1135 (2007).

93. J. R. Polimeni, B. Fischl, D. N. Greve, L. L. Wald, Laminar analysis of 7T BOLD using an imposed spatial activation pattern in human V1. *Neuroimage* **52**, 1334–1346 (2010).

94. S. Kashyap, D. Ivanov, M. Havlicek, B. A. Poser, K. Uludağ, Impact of acquisition and analysis strategies on cortical depth-dependent fMRI. *Neuroimage* **168**, 332–344 (2018).

95. G. J. Brouwer, D. J. Heeger, Decoding and reconstructing color from responses in human visual cortex. *J. Neurosci.* **29**, 13992–14003 (2009).

96. S. W. Provencher, Automatic quantitation of localized *in vivo* 1H spectra with LCModel. *NMR Biomed.* **14**, 260–264 (2001).

97. P. Bednařík, I. Tkáč, G. Federico, M. DiNuzzo, D. K. Deelchand, U. E. Emir, L. E. Eberly, S. Mangia, Neurochemical and BOLD responses during neuronal activation measured in the human visual cortex at 7 Tesla. *J. Cereb. Blood Flow Metab.* **35**, 601–610 (2015).

98. W. Bogner, S. Gruber, M. Doelken, A. Stadlbauer, O. Ganslandt, U. Boettcher, S. Trattnig, A. Doerfler, H. Stefan, T. Hammen, In vivo quantification of intracerebral GABA by single-voxel 1H-MRS-How reproducible are the results? *Eur. J. Radiol.* **73**, 526–531 (2010).

99. M. J. Donahue, J. Near, J. U. Blicher, P. Jezzard, Baseline GABA concentration and fMRI response. *Neuroimage* **53**, 392–398 (2010).

100. C. Sampaio-Baptista, N. Filippini, C. J. Stagg, J. Near, J. Scholz, H. Johansen-Berg, Changes in functional connectivity and GABA levels with long-term motor learning. *Neuroimage* **106**, 15–20 (2015).

101. J. Kolasinski, J. P. Logan, E. L. Hinson, D. Manners, A. P. Divanbeighi Zand, T. R. Makin, U. E. Emir, C. J. Stagg, A mechanistic link from GABA to cortical architecture and perception. *Curr. Biol.* **27**, 1685–1691.e3 (2017).

102. S. Kühn, F. Schubert, R. Mekle, E. Wenger, B. Ittermann, U. Lindenberger, J. Gallinat, Neurotransmitter changes during interference task in anterior cingulate cortex: Evidence from fMRI-guided functional MRS at 3 T. *Brain Struct. Funct.* **221**, 2541–2551 (2016).

103. A. D. Harris, N. A. J. Puts, R. A. E. Edden, Tissue correction for GABA-edited MRS: Considerations of voxel composition, tissue segmentation, and tissue relaxations. *J. Magn. Reson. Imaging* **42**, 1431–1440 (2015).

104. S. Quadrelli, C. Mountford, S. Ramadan, Hitchhiker's guide to voxel segmentation for partial volume correction of in vivo magnetic resonance spectroscopy. *Magn. Reson. Insights* **9**, 1–8 (2016).
